# Quantitative Multicolor Super-Resolution Microscopy Reveals Tetherin HIV-1 Interaction

**DOI:** 10.1371/journal.ppat.1002456

**Published:** 2011-12-15

**Authors:** Martin Lehmann, Susana Rocha, Bastien Mangeat, Fabien Blanchet, Hiroshi Uji-i, Johan Hofkens, Vincent Piguet

**Affiliations:** 1 Departments of Microbiology and Molecular Medicine, Dermatology and Venereology, University Hospital and Medical School of Geneva, Geneva, Switzerland; 2 Laboratory for Photochemistry and Spectroscopy, Department of Chemistry, Katholieke Universiteit Leuven, Heverlee, Belgium; 3 Department of Dermatology and Wound Healing, Cardiff University School of Medicine and University Hospital of Wales, Cardiff, Wales, United Kingdom; Universitätsklinikum Heidelberg, Germany

## Abstract

Virus assembly and interaction with host-cell proteins occur at length scales below the diffraction limit of visible light. Novel super-resolution microscopy techniques achieve nanometer resolution of fluorescently labeled molecules. The cellular restriction factor tetherin (also known as CD317, BST-2 or HM1.24) inhibits the release of human immunodeficiency virus 1 (HIV-1) through direct incorporation into viral membranes and is counteracted by the HIV-1 protein Vpu. For super-resolution analysis of HIV-1 and tetherin interactions, we established fluorescence labeling of HIV-1 proteins and tetherin that preserved HIV-1 particle formation and Vpu-dependent restriction, respectively. Multicolor super-resolution microscopy revealed important structural features of individual HIV-1 virions, virus assembly sites and their interaction with tetherin at the plasma membrane. Tetherin localization to micro-domains was dependent on both tetherin membrane anchors. Tetherin clusters containing on average 4 to 7 tetherin dimers were visualized at HIV-1 assembly sites. Combined biochemical and super-resolution analysis revealed that extended tetherin dimers incorporate both N-termini into assembling virus particles and restrict HIV-1 release. Neither tetherin domains nor HIV-1 assembly sites showed enrichment of the raft marker GM1. Together, our super-resolution microscopy analysis of HIV-1 interactions with tetherin provides new insights into the mechanism of tetherin-mediated HIV-1 restriction and paves the way for future studies of virus-host interactions.

## Author Summary

Human immunodeficiency virus 1 (HIV-1) assembles and interacts with cellular proteins at the plasma membrane of infected cells. Here, we analyzed individual HIV-1 virions, viral assembly sites and the mechanism of tetherin restriction by multicolor super-resolution microscopy using fully functional fluorescently labeled tetherin and viral proteins. Viral proteins within virions were visualized with nanometer resolution yielding new insight into the structure of the HIV-1. Our super-resolution analysis was extended to tetherin, a cellular restriction factor that inhibits the release of several enveloped viruses. Tetherin was localized in clusters of 70–90 nm at the plasma membrane that contain 5–11 dimers. In contrast tetherin clusters found at HIV-1 assembly sites contained on average 4–7 tetherin dimers. Clustering of tetherin was dependent on both tetherin membrane anchors. The transmembrane domain of tetherin associated with budding virions independently of GM1 lipid raft domains. Our data indicated that extended dimers tether HIV-1 virions directly to the cell. Overall, we provide for the first time super-resolution analysis of authentic virions, virus budding sites and HIV-1 interactions with the anti-viral factor tetherin. Our data offer novel insights into the mechanisms of tetherin restriction.

## Introduction

Although viruses heavily depend on the host cell machinery for their replication, they also face numerous blockades imposed by cellular proteins at several distinct steps in their life cycle. Recently, tetherin, an interferon-induced transmembrane protein has been shown to restrict the release of HIV-1 [1], [2] and other enveloped viruses [3]–[5]. Viruses also possess several anti-tetherin activities encoded by proteins such as HIV-1 Vpu [2], [6], SIV Nefs and Envelope (ENV) [7]–[9], HIV-2 ENV[10] and Karposi's sarcoma-associated herpesvirus K5 [5].

Tetherin possesses two membrane anchors in an unusual topology, namely a N-terminal transmembrane (TM) domain and a C-terminal glycophosphatidylinositol (GPI) lipid anchor, proposed to mediate lipid raft interaction [11]. The extracellular domains of two tetherin molecules form parallel cysteine-linked coiled-coil domains [12], [13]. Perez-Caballero *et al.* used tetherin mutants and artificial tetherin composed of fragments of heterologous proteins in a tetherin-like topology to demonstrate that tetherin inhibits HIV-1 release through direct tethering of virions to cells [13]. Direct incorporation of tetherin into HIV-1 virions was also confirmed by biochemical analysis and electron microscopy [13]–[15]. HIV-1 Vpu interacts with the tetherin transmembrane domain [6], [16] and counteracts tetherin by degradation and removal from the cell surface [2], [17]–[19]. Through these combined activities, Vpu impairs incorporation of tetherin into virions and restriction [13]. Detailed analysis of tetherin distribution in the plasma membrane, of the role of lipid rafts in HIV-1 tetherin interactions and of the orientation and number of tetherin molecules involved in restriction is still lacking.

HIV-1 assembly into virions of 100–150 nm diameter at the plasma membrane of infected cells involves an extensive range of host cell factors [20]. Widely used electron microscopy techniques provide detailed pictures of viral and cellular structures, but high density labeling of viral and cellular proteins as well as quantitative image analysis remain challenging. Novel single-molecule super-resolution imaging by photoactivated localization microscopy (PALM) [21], fluorescence PALM (fPALM) [22], stochastic optical reconstruction microscopy (STORM) [23] and direct STORM (dSTORM) [24] exploit photoswitching properties of photoactivatable fluorescent proteins (PAFP) and organic dyes to localize them with nanometer resolution. Multicolor super-resolution microscopy [25], [26] can resolve distances of 20–200 nm that are relevant for virus-host interactions and bridge the gap between Fluorescence Resonance Energy transfer (FRET) and conventional diffraction limited fluorescence microscopy [27].

Previous super-resolution imaging revealed single molecule dynamics and assembly of tandem-EosFP tagged HIV-1 Gag into virus-like particles of 100–200 nm [21], [28].

Here, we set up labeling of HIV-1 and tetherin with monomeric PAFP and antibody staining for multicolor super-resolution microscopy in cells. We visualized precise localization of HIV-1 proteins in virions and at budding sites at the plasma membrane of cells. Super-resolution analysis showed that tetherin formed clusters, whose integrity depended on both membrane anchors. Importantly, tetherin clusters closely associated with HIV-1 budding sites. Through a combination of biochemical analysis and super-resolution microscopy of tetherin mutants, we showed that tetherin restricts virion release as extended dimers and that the transmembrane domain of tetherin possesses affinity for HIV-1 assembly sites.

## Results

In order to perform single-molecule super-resolution microscopy of HIV-1, we fused different PAFP to HIV-1 Gag, the major structural protein of virions, yielding: Gag-Dronpa, Gag-PS-CFP2, Gag-Dendra2, Gag-mKikGR, Gag-mEosFP and Gag-PAmCherry (Figure S1A). The monomeric PAFP are expected to minimally interfere with HIV-1 particle formation [29], [30], a potential caveat of the Gag fused to tandem-EosFP. The different constructs were expressed together with full-length HIV-1 in 293T cells and analyzed for incorporation into HIV-1 particles, effect on viral infectivity and performance in single-color super-resolution imaging [31]. We selected Gag-Dronpa and Gag-mEosFP for super-resolution imaging of HIV-1 virions, due to their expression as full length fusion proteins, minimal impact on infectivity and superior signal-to-noise ratios (Figure S1B–D).

To visualize HIV-1 and cellular proteins labeled with PAFP or Alexa Fluor 647 labeled antibodies, we setup a two-color super-resolution microscope with widefield illumination in total internal reflection (TIR) mode. Fluorescence emission of Dronpa, mEosFP and Alexa Fluor 647 were detected simultaneously by two Electron Multiplying CCD cameras using specific filter sets and synchronized photoactivation/excitation/detection schemes as depicted in Figure S2. Notably, differences in alignment of both cameras and chromatic aberrations were corrected using a high resolution mapping procedure (Figure S3) based on local weighted mean transformation [32]. Colocalization precision of ∼17 nm throughout the total field of view was routinely achieved (Figure S3F). Our two-color super-resolution microscope therefore enabled colocalization analysis of proteins labeled with PAFP or antibodies in the range of 20–200 nm which covers scales relevant to HIV-1 host interactions.

### HIV-1 virion structure and assembly sites

To determine the localization of HIV-1 proteins within virions, we performed super-resolution microscopy on double-labeled HIV-1 particles.

**Figure 1:**
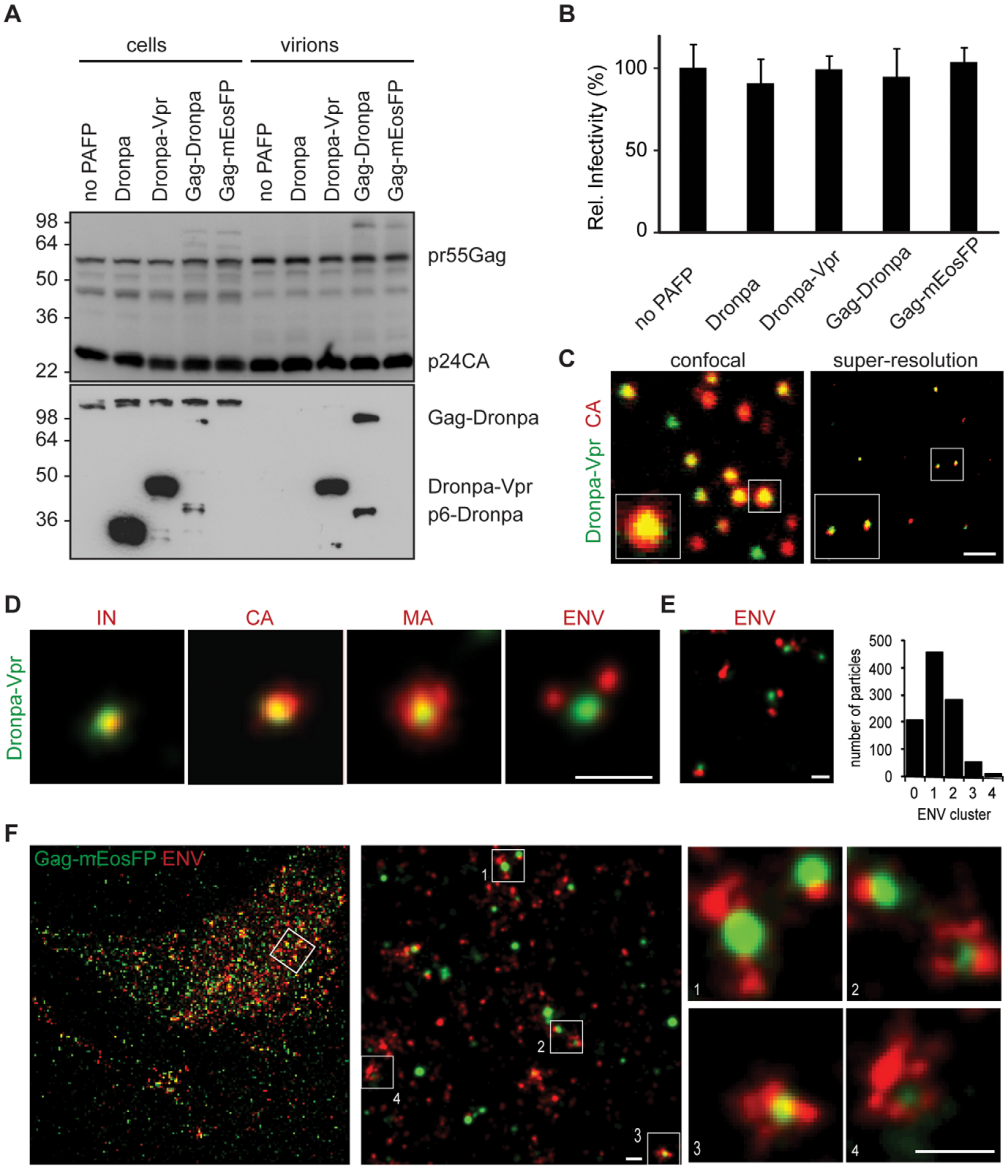
Structural features of single HIV-1 virions and assembly sites are revealed by multicolor super-resolution microscopy. (A) Western blot analysis (anti-p24CA upper, anti-Dronpa bottom) of cellular lysates and purified virions from 293T cells transfected with HIV-1 INHA and indicated photoactivatable fluorescent protein (PAFP). Sizes of molecular weight markers are shown in kilodaltons. (B) Relative infectivity of virions from (A). Error bars represent standard deviation of triplicate titrations. One representative experiment out of two is shown for panel A and B. (C) Virions labeled with Dronpa-Vpr and primary anti-CA and Alexa Fluor 647 secondary antibodies were analyzed by confocal laser scanning (left) or super-resolution microscopy (right). (D) Virions containing Dronpa-Vpr (green) were labeled by indirect immunofluorescence with primary antibodies against HA (IN), HIV-1 capsid (CA), matrix (MA) or gp120 (ENV) and Alexa Fluor 647 secondary antibodies (red). Representative super-resolution images from two virus preparations are shown. (E) Super-resolution image (left) of ENV (red) on Dronpa-Vpr particles (green). Quantification (right) was performed by counting the number of ENV clusters on 1000 HIV-1 particles in 4 images of two independent virus preparation. (F) HeLa cells expressing HIV-1 Δvpu and Gag-mEosFP (green) were labeled by indirect immunofluorescence with primary anti-gp120 (ENV) and Alexa Fluor 647 secondary antibodies (red), conventional resolution (left) and super-resolution image (right), scale bars 1 µm (c) and 200 nm (d–f).

HIV-1 virions specifically incorporated Dronpa-labeled Vpr, a HIV-1 protein associated with viral cores, Gag-Dronpa and Gag-mEosFP, but not Dronpa alone (Figure 1A and Figure S4A). Notably, labeling HIV-1 virions with the monomeric PAFP fusions minimally affected infectivity and therefore likely preserves virion structure (Figure 1B).

HIV-1 virions that contained HA-tagged integrase (INHA) and Dronpa-Vpr were fixed on coverslips, permeabilized and further labeled by indirect immunofluorescence against Integrase (IN), capsid (CA), matrix (MA) or gp120 envelope (ENV) followed by Alexa Fluor 647-coupled secondary antibodies (Figure 1C–E). Super-resolution microscopy of double labeled virus particles showed an important increase in resolution when compared to diffraction-limited confocal laser scanning or total internal reflection fluorescence (TIRF) microscopy (Figure 1C and Figure S1D). The high degree of colocalization in super-resolution images of the two HIV-1 proteins Dronpa-Vpr and CA (Figure 1C) demonstrated the performance of the calibration procedure in a biological context. The sizes of HIV-1 structures were determined through cluster analysis of single molecule localizations as described in Materials and Methods after testing on simulated clusters (Figure S5). We found similar average sizes for Dronpa-Vpr (94±17 nm), Gag-Dronpa (108±39 nm) and Gag-mEosFP (94±37 nm, mean±StD) consistent with their localization inside HIV-1 virions (Figure S4B).

In contrast, IN, CA, MA and ENV showed more variable sizes: 75±20 nm for IN, 112±31 nm for CA, 117±45 nm for MA and 127±45 nm for ENV (mean±StD, Figure 1D and Figure S4C). Notably, IN colocalized with Dronpa–Vpr cores as a discrete structure with narrow size distributions, likely due to their common presence in the viral core. Similar sizes observed for CA and MA structures could result from proximity of the C-terminal epitope of mature MA and the epitope recognized by the CA antibody. In contrast, HIV-1 ENV was found in 1-2 discrete peripheral clusters per Dronpa-Vpr containing virion (Figure 1E).

Next, we used super-resolution microscopy to visualize HIV-1 in a cellular context. We analyzed HIV-1 protein distribution at the plasma membrane of non-permeabilized HeLa cells transfected with HIV-1 and Gag-mEosFP and stained for ENV. We observed distinct clusters of Gag-mEosFP surrounded by antibody labeled ENV clusters (Figure 1F) that represent HIV-1 assembly sites [21], [28], [29]. Altogether, our data demonstrates that super-resolution microscopy allows precise localization of HIV-1 proteins in infectious virions and budding structures, which previously could only be observed by electron microscopy [33]–[35].

### Tetherin distribution and orientation

**Figure 2:**
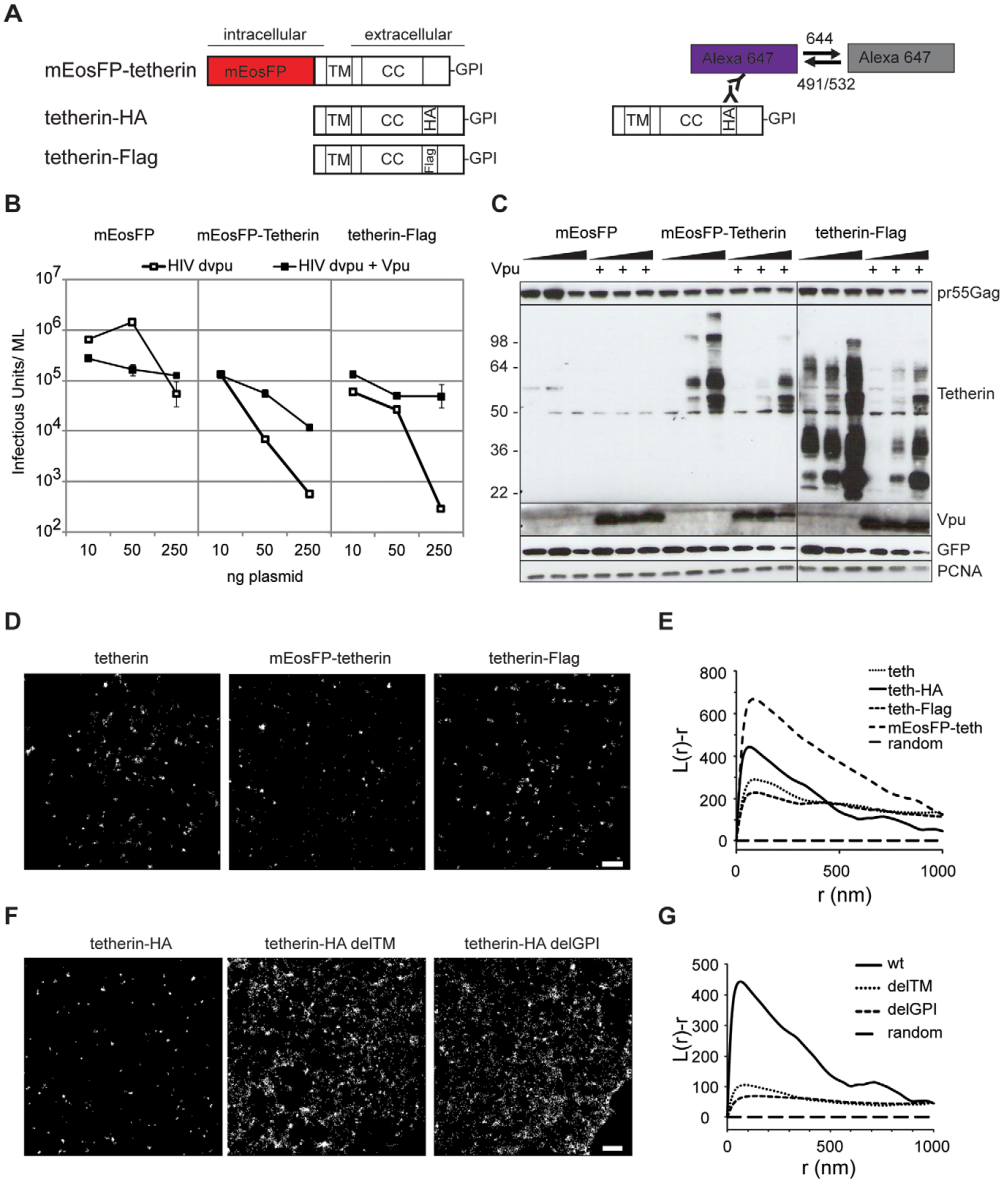
Tetherin localization to micro-domains depends on both membrane anchors. (A) Schematic presentation of tetherin constructs used and labeling/photoswitching scheme of fluorescent Alexa Fluor 647 (violet) and dark state (grey), tetherin structural features are: transmembrane domain (TM), coiled-coil domain (CC) and Glycosylphosphatidylinositol (GPI) anchor. HA or Flag are internal HA or Flag tags. (B) 293T cells were transfected with HIV-1 Δvpu and either mEosFP, mEosFP-tetherin or tetherin-Flag without or with Vpu as indicated and infectious output was determined on HeLa indicator cells. Error bars represent range of duplicate titrations. (C) Western blot analysis of cell lysates from B) was performed for pr55Gag, tetherin, Vpu, GFP as transfection control and PCNA as loading control. Sizes of molecular weight markers are shown in kilodaltons. One representative experiment out of two is shown for panel B and C. (D) Representative regions of super-resolution images of HeLa cells expressing HIV-1 Δvpu and empty plasmid, mEosFP-tetherin or tetherin-Flag that were labeled by indirect immunofluorescence against tetherin or Flag or left unlabeled (mEosFP-tetherin), scale bar 500 nm. E) Ripley's L analysis: normalized L(r)-r plots indicate clustering at distances r with positive L(r)-r values, F) Representative regions of super-resolution images of HeLa cells expressing HIV-1 Δvpu and indicated tetherin-HA or mutant constructs. The cells were labeled by indirect immunofluorescence with primary anti-HA and Alexa Fluor 647 secondary antibodies, scale bar 500 nm. G) Ripley's L analysis indicates higher degree of clustering of wt tetherin than tetherin mutants delTM and delGPI.

We next set out to visualize HIV-1 tetherin interactions at the plasma membrane using this technique. First, the plasma membrane distribution of endogenous and overexpressed tetherin was analyzed by super-resolution microscopy in cells. Tetherin constructs containing N-terminal mEosFP or epitope-tags (HA or Flag) inserted after the extracellular coiled-coil domain (Figure 2A) efficiently restricted the release of vpu-deficient HIV-1 (HIV-1 Δvpu) and are counteracted by Vpu in transfected 293T cells (Figure 2B and Figure S6A). Notably the cellular levels of mEosFP-tetherin, tetherin-Flag and tetherin-HA were reduced in presence of HIV-1 Vpu (Figure 2C and Figure S6B), indicating efficient Vpu-mediated degradation of these constructs. The N-terminal fusion of mEosFP to tetherin does not interfere with HIV-1 assembly (Figure S6C) and no cleavage of the fusion protein was observed (Figure 2C and Figure S6C). Overall, labeling tetherin with PAFP and epitope-tags minimally affects restriction activity and preserves Vpu-sensitivity.

In HeLa cells we found endogenous and overexpressed tetherin in homogenously distributed clusters of 70–90 nm using labeling with mEosFP or antibody staining followed by super-resolution microscopy (Figure 2D). Ripley's L function was previously used to characterize extent of clustering of influenza hemagglutinin (HA) and T cell receptor complexes [36], [37] and was tested on simulated clusters of 50–400 nm (Figure S5). Ripley's L function indicated clustering of endogenous tetherin and all tetherin constructs tested (Figure 2E). In contrast tetherin mutants lacking the transmembrane domain (delTM) or GPI anchor (delGPI) showed decreased clustering (Figure 2F), which was confirmed by decreased peaks of Ripley's L function that shifted to larger distances compared to wild-type tetherin (Figure 2G). Overall, labeling of tetherin for super-resolution microscopy preserved restriction activity, Vpu-sensitivity and revealed a clustered distribution, which depends on both tetherin's membrane anchors.

### Distribution of tetherin molecules at HIV-1 assembly sites

**Figure 3:**
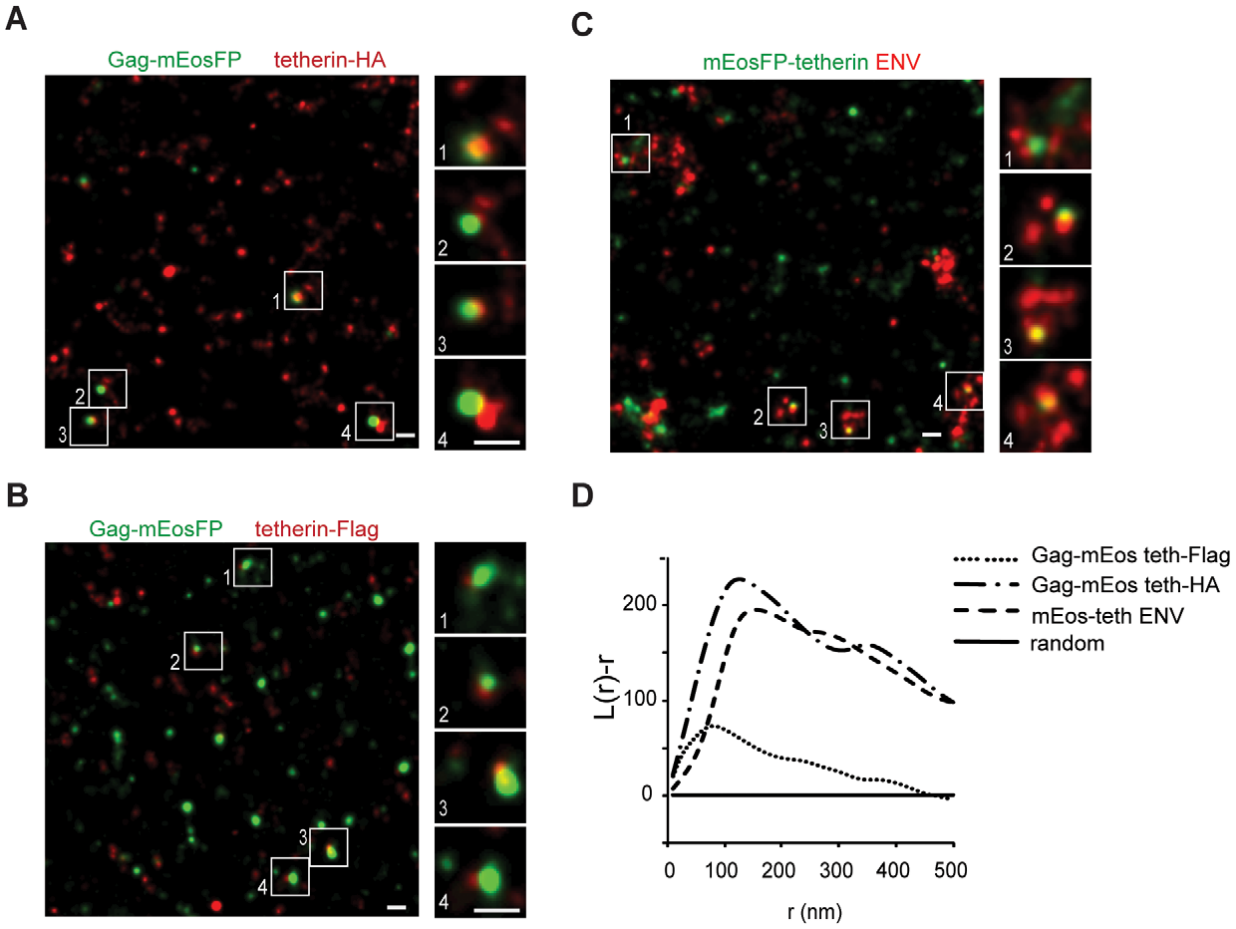
Single tetherin domains cocluster with HIV-1 budding sites. (A–C) Representative regions of super-resolution images of HeLa cells transfected with HIV-1 Δvpu and: (A) Gag-mEosFP and tetherin-HA; (B) Gag-mEosFP and tetherin-Flag and (C) mEosFP-tetherin. Tetherin-HA, tetherin-Flag and HIV-1 ENV were stained by indirect immunofluorescence for HA, Flag and ENV, respectively. (D) Bivariant Ripley's L analysis indicates coclustering of tetherin-HA, tetherin-Flag and mEosFP-tetherin with HIV-1 Gag-mEosFP and ENV, respectively. Images are representative of 5-10 cells from two independent transfections.

Since super-resolution microscopy enabled us to resolve structural features of viral particles and the distribution of tetherin (Figures 1 and 2), we next determined where tetherin localizes with respect to HIV-1 assembly sites. HeLa cells were cotransfected with HIV-1 Δvpu and Gag-mEosFP and tetherin-HA or tetherin-Flag and analyzed by indirect immunofluorescence and super-resolution microscopy. Gag-mEosFP-containing budding structures at the plasma membrane were mostly found in close proximity with a single cluster of tetherin-HA or tetherin-Flag (Figure 3A and B). Similarly, mEosFP-tetherin clusters were found close to groups of HIV-1 ENV clusters at HIV-1 assembly sites (Figure 3C). Close examination of 500 individual tetherin-positive budding sites revealed that 80% contained a single cluster of mEosFP-tetherin (Figure S6D). Finally bivariant Ripley's L function confirmed coclustering of different tetherin constructs with either Gag-mEosFP or HIV-1 ENV at HIV-1 budding sites (Figure 3D). We conclude that tetherin in single clusters closely associates with HIV-1 budding sites.

### Tetherin restriction mechanism

**Figure 4:**
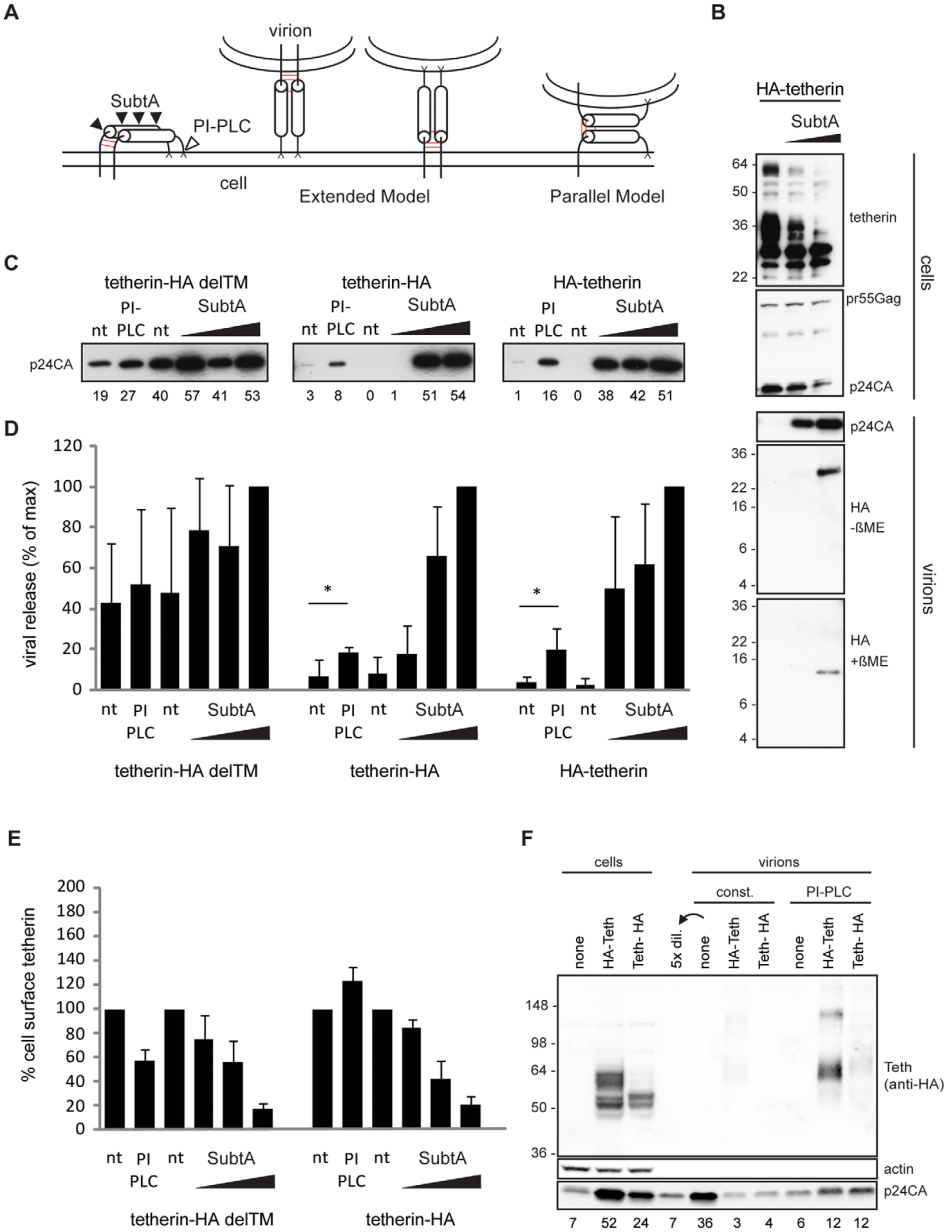
Tetherin restricts HIV-1 release as extended dimers. (A) Model of tetherin, enzymatic cleavage sites of subtilisin A (SubtA) and phosphatidyl-inositol-specific phospholipase C (PI-PLC) and possible orientations during restriction, disulfide bonds are represented in red. (B) Western blot analysis of subtilisin A stripped cells and virions. 293T cells transfected with HIV-1 Δvpu and HA-tetherin were treated with subtilisin A (0,5 or 50 µg/ML). Released Virions were pelleted through sucrose and analyzed under non-reducing (-βME) or reducing (+βME) SDS-PAGE/Western blot together with corresponding cell lysates (+βME). Data is representative of 2 experiments. (C) Western blot analysis (anti-p24CA) of purified virions released from non-treated (nt), PI-PLC (1 U/ML) or subtilisin A (0.2, 1 or 5 µg/ML) treated 293T cells transfected with HIV-1 Δvpu, GFP and either transmembrane-deficient tetherin-HA delTM, tetherin-HA or HA-tetherin, that contain either a C or N-termal HA-tag. Numbers below each lane indicate integrated densities in arbitrary units and are representative of 3 experiments. (D) Quantification of viral release from non-treated (nt), PI-PLC or subtilisin A treated cells 3-4 independent experiments as in B), maximal release by 5 µg/ML Subtilin A treatment was normalized to 100%. Error bars represent standard deviations and * indicates statistically significant difference with p = 0.05 (two-tailed paired Student's t-test). (E) Enzymatic removal of tetherin from cells in (C) was analyzed by Fluorescence-activated cell sorting (FACS). Shown are mean fluorescence intensities of anti-HA labeling in GFP-positive cells with non-treated cells set to 100%. Error bars represent standard deviations (n = 3). (F) Western blot analysis of PI-PLC stripped virions from 293T cells transfected with HIV-1 Δvpu and HA-tetherin or tetherin-HA. Virions that were constitutively released (const.) or released following incubation of cells with 1 U/ML PI-PLC were pelleted through sucrose. Virions and corresponding cell lysates were analyzed by Western blotting with anti-HA, anti-p24CA and anti-actin antibodies. Numbers below each lane indicate integrated densities of p24CA in arbitrary units. Sizes of molecular weight markers are shown in kilodaltons.

As both tetherin membrane anchors are essential for restricting HIV-1 release [13], different orientations of tetherin dimers across the cell and viral membrane are possible. Two models must be considered: (i) the “extended model” in which pairs of membrane anchors are incorporated into the cell membrane and the viral membrane, and an extended coiled-coil domain spans the gap between both membranes or (ii) the “parallel model” where one tetherin monomer is incorporated into the cell membrane and the other monomer into the viral membrane (Figure 4A). The apparent distances of circa 17 nm found between tethered virions by electron microscopy [6] and the structure of the tetherin ectodomain [12] favor the extended model but definitive proof is missing.

Tetherin-restricted virions are efficiently released from the cell surface by treatment with subtilisin A, a protease with relatively low specificity [1], [6], [14], [15]. If virions are retained following the parallel model, subtilisin A treatment should leave monomeric low molecular weight N-termini inside stripped particles.

293T cells expressing HIV-1 Δvpu and N-terminal HA-tagged tetherin were treated with subtilisin A, cell lysates and released virions were then analyzed by Western blot. Analysis of cellular extracts showed constant pr55Gag content, whereas the majority of glycosylated tetherin at 36 and 60 kDa, as well as virions containing p24CA were efficiently removed by subtilisin A treatment (Figure 4B). Only a HA fragment of ∼26 kDa was found inside stripped virions by non-reducing SDS-PAGE/Western blot analysis (Figure 4B). Under reducing SDS-PAGE a single band migrated at ∼13 kDa, consistent with a previously proposed cleavage site at RNVT/H68 [13], [14]. Altogether, we found dimeric N-termini associated with subtilisin A stripped virions, which is not compatible with the parallel model of tetherin orientation.

If tetherin retains viruses via the extended model, cleavage of the tetherin GPI anchor by phosphatidyl-inositol-specific phospholipase C (PI-PLC) should release tethered virions. To test this hypothesis, 293T cells transfected with HIV-1 Δvpu and tetherin-HA, HA-tetherin or the inactive mutant tetherin-HA delTM (Figure S6A and [13]) were treated with PI-PLC or subtilisin A. Stripped virions were pelleted through sucrose and analyzed by Western blot. Both PI-PLC and subtilisin A treatment released virions retained by tetherin (Figure 4C). Quantification from several experiments revealed that PI-PLC treatment (1 U/ML) released 20% of virions compared to the maximal release by 5 µg/ML subtilisin A (Figure 4D). To compare PIPLC and subtilisin A activities on tetherin we used wt tetherin HA and the mutant delTM, that is attached to the cell only via a GPI anchor. Fluorescence-activated cell sorting (FACS) analysis revealed that PI-PLC specifically reduced cell-surface levels of tetherin-HA delTM, whereas increased signal from tetherin-HA could result from increased access of antibodies to the internal HA tag after tetherin GPI cleavage (Figure 4E). Subtilisin A treatment removed the majority of tetherins from the cell surface, indicating that lower release of tetherin restricted virions by PI-PLC can be explained by its lower activity in cleaving the tetherin GPI anchor when compared to proteolysis cleavage by subtilisin A.

Finally, virions released by PI-PLC treatment contained nearly full length dimeric tetherin (Figure 4F). Therefore, since both PI-PLC and subtilisin A treatments removed tethered virions, which respectively contained dimeric tetherin and dimeric N-terminal tetherin fragments we conclude that tetherin restricts HIV-1 release as an extended dimer.

**Figure 5:**
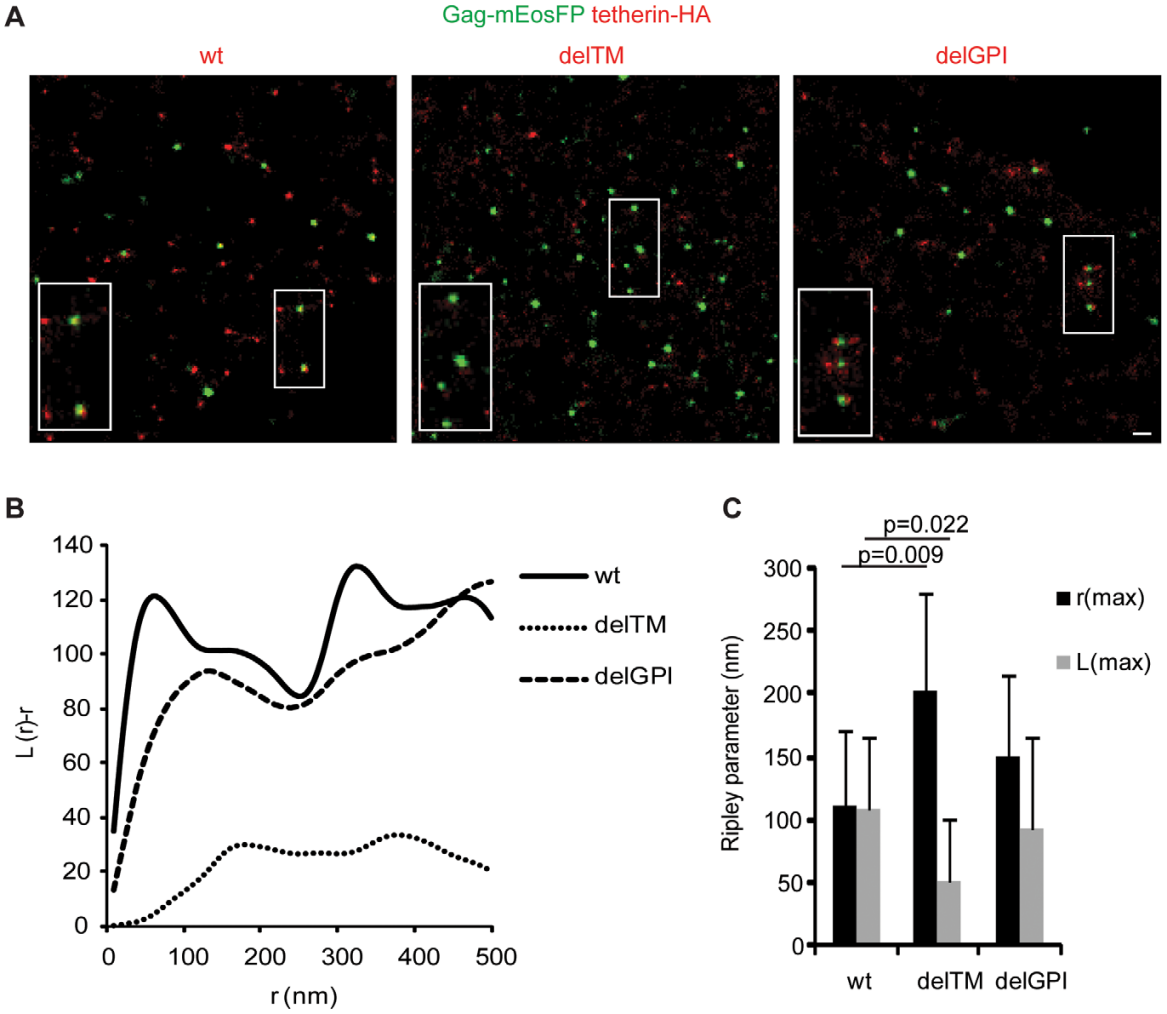
Association of tetherin with HIV-1 budding sites depends on its transmembrane domain. A) Representative regions of super-resolution images of HeLa cells transfected with HIV-1Δvpu, Gag-mEosFP and tetherin-HA wt, delTM or delGPI, scale bar 200 nm, B) bivariant Ripley's L analysis on fields shown in A), C) Multiple fields were analyzed by bivariant Ripley's L function and r(max) and L(max) determined, error bars represent standard deviations (9 fields from 3 cells per conditions), p values were determined by Student's t-test.

To characterize further the orientation of tetherin upon incorporation into the membrane of assembling virions, we analyzed the distribution of tetherin mutants lacking one of the membrane anchors relative to HIV-1 budding sites by super-resolution microscopy and co cluster analysis using bivariant Ripley's L function. HeLa cells were transfected with HIV-1 Δvpu, Gag-mEosFP and tetherin-HA or its mutants delTM and delGPI and labeled by anti-HA immunofluorescence. Tetherin-HA and the delGPI mutant, but not delTM associated with Gag-mEosFP-containing budding sites (Figure 5), indicating that the tetherin transmembrane domain drives tetherin localization to HIV-1 budding sites.

**Figure 6:**
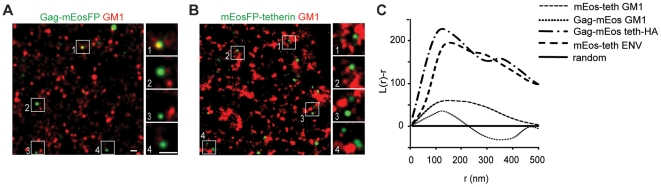
Tetherin domains and HIV-1 budding sites do not overlap with GM1 containing lipid rafts. (A,B) Representative regions of super-resolution images of HeLa cells transfected with HIV-1 Δvpu and Gag-mEosFP(A) or mEosFP-tetherin (B) that were fixed and stained for GM1 with Cholera toxin B Alexa Fluor 647, scale bars 200 nm (C) Bivariant Ripley's L analysis of panel A and B of this figure and Figure 3A and C Images and cocluster analysis shown is representative of multiple fields from 4–6 transfected cells from two independent transfections.

Finally to test whether tetherin could associate with budding structures via lipid rafts, HeLa cells expressing HIV-1 Δvpu and Gag-mEosFP or mEosFP-tetherin, were fixed, stained for the lipid raft marker GM1 using Alexa Fluor 647 Cholera-toxin and analyzed by super-resolution microscopy and co cluster analysis using bivariant Ripley's L function. GM1 localized to clusters of variable sizes that showed minor coclustering with mEosFP-tetherin domains, but did not show significant overlap (Figure 6B and C), indicating that both proteins are found in distinct, but adjacent domains. In contrast, we could not detect significant coclustering of GM1 with Gag-mEosFP-containing HIV-1 budding sites (Figure 6A and C). As positive control for coclustering, we monitored mEosFP-tetherin and tetherin-HA at HIV-1 budding sites labeled by HIV-1 ENV and Gag-mEosFP, respectively (Figure 3D and 6C). In addition, the GPI anchor was not sufficient to mediate enrichment of tetherin-HA delTM at budding sites (Figure 5). We conclude that tetherin and GM1, although both were reported to associate with lipid rafts, localize to different but adjacent lipid domains and that GM1-containing lipid rafts and the tetherin GPI anchor are unlikely to drive tetherin to HIV-1 assembly sites.

### Quantification of tetherin molecules at HIV-1 assembly sites

**Figure 7:**
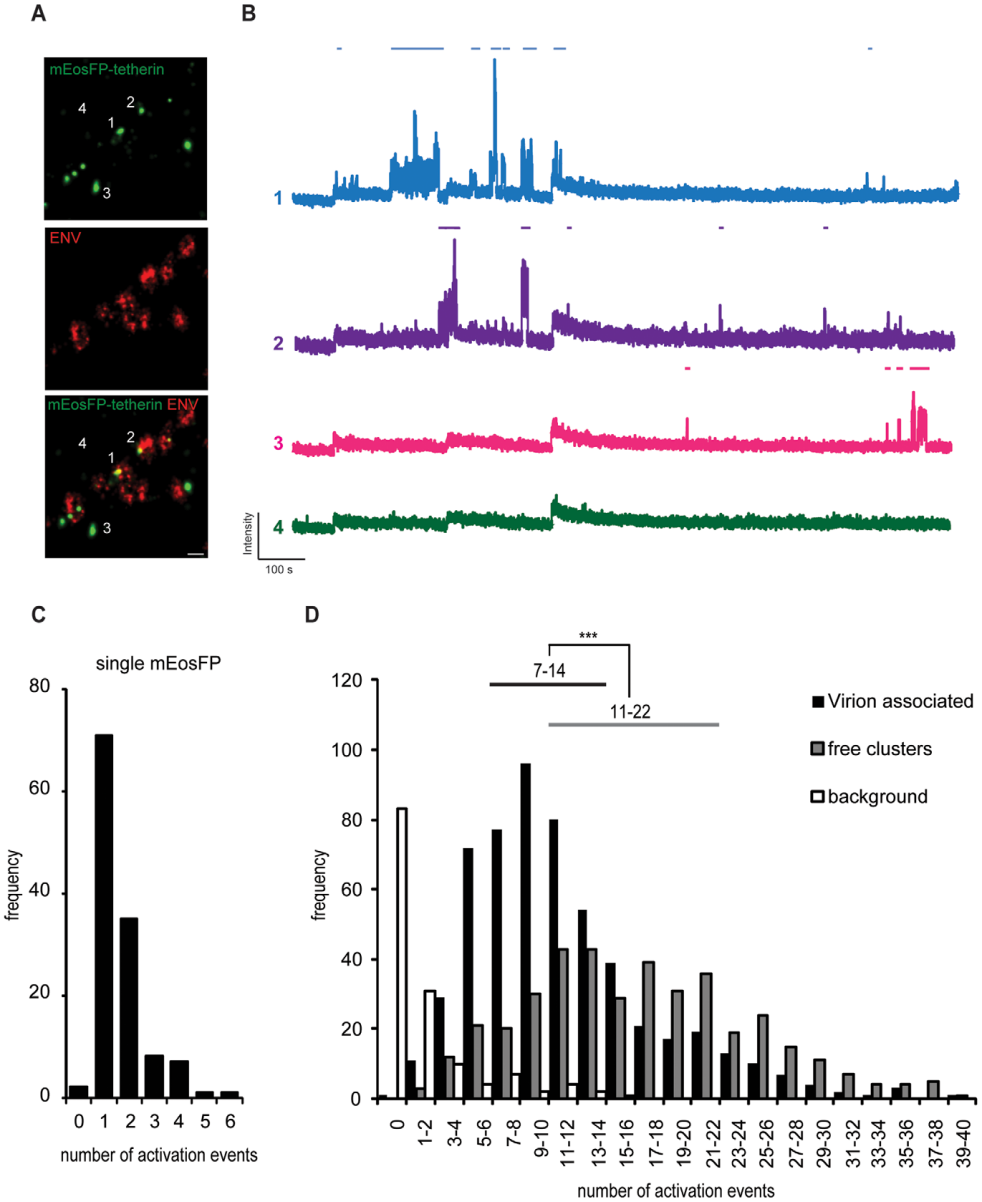
Single fluorescent molecule quantification of mEosFP-tetherin at HIV-1 budding sites. (A) Representative region of super-resolution images of HeLa cells transfected with HIV-1 Δvpu and mEosFP-tetherin and stained by indirect immunofluorescence for HIV-1 ENV. Acquisition was performed under alternating 561 and 644 nm excitation and continuous 405 nm photoactivation for 15 000 frames, scale bar 200 nm. (B) Intensity traces of single mEosFP-tetherin clusters as depicted in (A). Automated detection of activation events on top of each trace is depicted using a threshold of 10 standard deviations above background and a dark time of 5 s. (C) Histogram of number of activation events per single mEosFP molecules in 1% PVA (n = 125). (D) Histogram of number of activation events of mEosFP-tetherin molecules in free clusters (n = 400) or clusters that are associated with HIV-1 budding sites (n = 550) in 5 cells. Background was selected in regions without clusters. Range indicates 25th–75th percentile. *** indicates statistically significant difference with p<0.001 (two-tailed paired Student's t-test).

Single-molecule imaging and localization of PAFP provides super-resolution images and was previously used to estimate molecule numbers [37]–[39]. Detailed photophysical characterization of the irreversible photo-convertible mEos [40] revealed long-lived dark states of the photoactivated red form that could lead to clustering artifacts and significant overcounting [39], [41]. Annibale *et al.* showed that continuous instead of pulsed photoactivation by 405 nm light and the introduction of dark times significantly reduced overcounting [41]. Following this methodology, fields of monodispersed mEosFP were used to determine appropriate imaging and analysis parameter including 561 nm excitation intensity [42], continuous 405 nm photoactivation and a dark time of 5 s. As a result, we found 1–2 reactivation events per single mEosFP molecule (25th–75th percentile range, Figure 7C and Figure S7), which may reflect the presence of a minor fraction of mEosFP multimers. Subsequently, using this optimized imaging and analysis parameters we estimated the number of mEosFP-tetherin molecules in free clusters and at HIV-1 budding sites (Figure 7A,B). At 550 assembly sites, we found 7–14 mEosFP-tetherin molecules (25th–75th percentile range, median is 10), corresponding to 4–7 tetherin dimers per budding site (Figure 7D). In contrast 400 mEosFP-tetherin clusters in the absence of HIV-1 contained 11-22 mEosFP-tetherin molecules (25th–75th Percentile range, median is 16), corresponding to 5–11 tetherin dimers (Figure 7D). The difference is significant (p<0.001, Student's t-test) and indicates that tetherin clusters can associate with HIV-1 budding sites. Approximately 70% of tetherin molecules within a cluster stay associated with budding virions and may participate in restricting HIV-1 release.

## Discussion

We have performed biochemical and super-resolution analysis to provide further understanding of the interactions between the restriction factor tetherin and HIV-1.

First, to achieve specific and high density labeling of HIV-1 for super-resolution microscopy, we tested different monomeric PAFP in HIV-1 Gag fusions, similar to Gag-GFP that showed budding at the plasma membrane [29], [30]. Gag-Dronpa, Gag-mEosFP and Dronpa-Vpr were incorporated into full HIV-1 particles, minimally affected infectivity and enabled super-resolution microscopy analysis of virions. This confirmed the expected virion diameter of 100 nm. Of note is the fact that Gag, fused to 50 kDa dimeric Eos protein, produced larger virus-like particles of 100–200 nm in initial super-resolution studies [21], [28]. For multicolor super-resolution, we labeled HIV-1/cellular proteins with Dronpa, mEosFP or antibodies coupled to the photoswitchable dye Alexa Fluor 647 [24], [43]. Using simultaneous detection of two fluorescent markers and high-resolution registration mapping [32] we acquired two-color super-resolution images within few minutes and with a colocalization precision of 17 nm throughout a field of view. The precise localization of antibody-labeled viral proteins within Dronpa-Vpr virions was determined by super-resolution microscopy. In accordance with PALM and electron microscopy [33] structures labeled with CA or MA antibodies had sizes of 112 nm and 117 nm, respectively and colocalized with Dronpa-Vpr. In contrast, HIV-1 integrase localized to a structure with a characteristic size of 75 nm within Dronpa-Vpr containing cores. HIV-1 ENV was predominantly found in 1–2 peripheral clusters close to Dronpa-Vpr, consistent with their localization in the viral membrane. A clustered distribution of ENV on HIV-1 virions was observed in electron tomography images and could be functionally relevant during cell attachment and fusion [35], [44].

HIV-1 assembly at the plasma membrane of HeLa cells was previously visualized using diffraction-limited microscopy [29], [30], [45]. Super-resolution microscopy of Gag-mEosFP and antibody-stained ENV on non-permeabilized cells revealed important structural features of budding sites. Gag-mEosFP clusters of varying sizes together with clustered ENV were found in the same TIRF imaging plane close to the coverslip, indicating assembly at the plasma membrane. As previously noted, ENV was found in distinct clusters [46] that incorporated into Gag-mEosFP assembly sites. Overall, our super-resolution microscopy analysis provides a detailed picture of HIV-1 virions and their assembly sites, which was previously exclusively obtained by electron microscopy.

We applied super-resolution microscopy for the HIV-1 cellular restriction factor, tetherin. Endogenous and overexpressed tetherin were previously identified in endosomal compartments and in clusters at the plasma membrane both by fluorescence microscopy [2], [3], [12], [15] and electron microscopy [14], [15], [47]. The overall clustered distribution of tetherin was not influenced by HIV-1 particle formation, however localization of tetherin at budding sites was noted in some cases [2], [3], [14], [15], but not all [18], [48]. The size of the clusters could not be determined due to the diffraction-limited resolution of conventional fluorescence microscopy or low density labeling in immuno-electron microscopy.

Using different labeling approaches for super-resolution microscopy and calibrated size measurements we found that both endogenous and overexpressed tetherins are organized in 70–90 nm clusters at the plasma membrane of HeLa cells. Annibale *et al* recently reported that dark state recovery of mEos2 can cause clustering artifacts, due to repeated localization of single molecules [41]. Since we observe similar cluster sizes for all tetherin constructs and endogenous tetherin, consistent clustering of mEosFP-tetherin using comparable imaging parameter as [41] and a significant decrease in clustering of different mutant tetherins we report here a relevant localization of tetherin at the plasma membrane.

Notably, our increased resolution revealed that both membrane anchors of tetherin are required for clustering possibly through high order complexes between dimers [13], interaction of the cytoplasmic tail with the actin cytoskeleton [49] and lipid raft association via the GPI anchor [11].

Several models depicting the orientation of tetherin during the restriction on release of enveloped viruses have been proposed [6]. Here, we provide biochemical and microscopic evidence for HIV-1 restriction via an extended conformation of tetherin dimers. This model requires that pairs of membrane anchors incorporate into the cell membrane and the viral membrane and an extended coiled-coil domain spans the gap between both membranes (Figure 4). PI-PLC and subtilisin A treatment of 293T cells transfected with tetherin efficiently removed virions from the surface that contained dimeric tetherins. Of note, vpu-deficient HIV-1 virions were not released by PI-PLC treatment from HeLa cells, but efficient cleavage of the tetherin GPI anchor was not demonstrated [15], [50]. Furthermore, slower enzymatic cleavage of the GPI anchor could occur in virion associated clusters of tetherin compared to efficient proteolytic cleavage by subtilisin A. In HeLa cells higher endocytosis rates could result in lower amounts of vpu-deficient virions bound to the cell surface compared to 293T cells [1]. Virus accumulation in biofilm-like extracellular assemblies [51] could further limit stripping efficiency by PI-PLC. Alternatively a fraction of tetherin that contains a second transmembrane domain instead a C-terminal GPI-anchor would be insensitive to PI-PLC treatment [50]. Since our results and previous reports indicate a C-terminal GPI-modification of rat and human tetherin [11], [13], [52] further biochemical analysis of tetherin C-terminal membrane anchor by mass spectroscopy is needed.

Of note, our biochemical analysis does not provide information on initial orientation of extended tetherin dimer at assembly sites since chains and clusters of tethered HIV-1 particles contain both of the possible orientations (Figure 4A) [13].

Therefore, we compared the incorporation of tetherin mutants lacking either the TM or GPI membrane anchor into single budding sites in HeLa cells by super-resolution microscopy. We found that the tetherin transmembrane domain stably associated with HIV-1 membranes during assembly.

Alternatively tetherin could also associate with HIV-1 budding sites via shared localization to lipid raft domains as both show some resistance to cold detergent extraction [1], [11], [53], [54]. Both tetherin and Gag also cofractionated with the lipid raft marker caveolin [55]. Nevertheless cofractionation of proteins with raft markers does not prove their direct association or localization to similar lipid raft domains [56]. Indeed crosslinking antibodies against tetherin inhibited its antiviral effect but increased tetherin/Gag cofractionation [55].

Using super-resolution microscopy we found tetherin clusters in close association with GM1 lipid domains, but without significant overlap as previously noted for other raft proteins [57]. HIV-1 budding sites did not show significant association with GM1 lipid domains or with a tetherin mutant containing only the GPI membrane anchor. Recently HIV-1 Gag multimerization was shown to induce the coalescence of lipid raft markers and tetraspanins as visualized by antibody copatching, FRET analysis and single molecule tracking [58], [59]. Both studies required copatching of raft markers (GM1, CD55 and HA–TM) and tetraspanins (CD9, CD81) to demonstrate their enrichment at HIV-1 budding sites due to the limited detection sensitivity of conventional fluorescence microscopy. Copatching could affect protein mobility and association with HIV-1 budding sites. GFP-GPI and unpatched CD55 failed to stably associate with viral assembly sites [45], [59]. The later observations are consistent with our results obtained from unpatched GM-1 that was stained after fixation. Additionally the stable association of patched HA-TM and tetraspanins with viral assembly sites [58], [59] is in line with our observation that clustered tetherin molecules stably associates with the viral membrane via their transmembrane domains.

Altogether the tetherin GPI modification and the association with GM1 lipid domains are not important for localization of tetherin to HIV-1 budding sites, but implicated in local concentration of tetherin (Figure 2) and maybe important for the endocytosis of retained virions. Alternatively tetherin could associate with HIV-1 assembly sites via other lipid rafts or tetraspanin-enriched domains. More efficient tetherin restriction could be obtained via late and stable incorporation into budding membranes within tetraspanin-enriched domains compared to early or transient association observed with raft markers [58], [59].

At HIV-1 assembly sites, we found single tetherin clusters. mEosFP-tetherin showed a clustered plasma membrane distribution, restricted efficiently HIV-1 particle release in a Vpu-dependent manner (Figure 2A,B and Figure S6) and did not interfere with Gag assembly (Figure S6A). Therefore, the irreversible photoswitching properties of mEosFP allowed us to determine relevant mEosFP-tetherin quantities at HIV-1 assembly sites. Careful photo-physical characterization of purified mEosFP and the adjustment of imaging and analysis parameters enabled reliable single molecule counting of mEosFP.

We found 5–11 tetherin dimers in single clusters in the absence of HIV-1 and 4–7 tetherin dimers associated with HIV-1 budding sites, that represents a significant difference (p<0.001, Student's t-test). This indicates that about 70% of tetherin molecules within a cluster remain stably associated with budding sites possibly through incorporation of their TM domains into the viral membrane. Overall a low number of clustered tetherin dimers is sufficient to restrict the release of newly formed virions. Interestingly crosslinking antibodies against tetherin interfere with tethering function, reduce incorporation of tetherin into virions and affected the distribution of tetherin within membrane raft fractions [55]. Therefore it is possible that antibody crosslinking affects tetherin clustering, but cannot be detected by conventional diffraction limited microscopy [55]. We propose that the supramolecular organization of tetherin dimers in clusters could concentrate and position tetherin for optimal restriction and limit access to viral countermeasures. In summary, our biochemical and super-resolution analysis provided new insights into tetherin interaction with HIV-1 virions. We can propose the following mechanism for HIV-1 restriction by tetherin: Initially, tetherin locally concentrates in clusters containing 5–11 dimers and this involves both TM and GPI membrane anchors. In the absence of Vpu, tetherin N-termini associate with HIV-1 budding sites independently of GM1-enriched raft domains and become trapped during Gag-multimerization. Clusters containing 4 to 7 tetherin dimers remain associated with budding virions and can mediate restriction. Flexible coiled-coil interactions within dimers [12] are likely to enable retention of GPI anchors within the host cell membrane during budding and membrane scission. The final predicted topology is that of extended tetherin dimers with N-termini inside newly formed virions.

Our study demonstrates that multicolor super-resolution imaging allows characterization of the interplay between viral and cellular structures with nanometer resolution. Quantitative analysis of single molecule localization in combination with biochemical analysis offers novel insights into the tetherin restriction mechanism and can be used to investigate virus-host interactions.

## Materials and Methods

### Cell lines

Human cell lines 293T, HeLa and HeLa-derived TZMbl were obtained through NIH AIDS Research and Reference Reagent Program and grown under standard conditions.

### Plasmids and reagents

The HIV-1 expression vectors pR9 INHA (kind gift of F.D. Bushman, University of Pennsylvania) and vpu-deficient pR9 Δvpu (HIV-1 Δvpu) are based on pR9 [60]. Plasmids coding for Gag-PAFP were obtained by replacing GFP from pGag-GFP [61] with Dronpa [62], mKikGR [63], mutant mKikGR15.1 containing K141E and V160I (kind gifts from A. Miyawaki, Riken Brain Science Institute, Japan), PS-CFP2, Dendra2 [64] (both from Evrogen), mEosFP [40] (pQE32 mEosFP was a kind gift of J.Wiedenmann, University of Southampton, U.K) or PAmCherry1 [65] (kind gift of V. Verkhusha, Albert Einstein College of Medicine, NY) by standard PCR cloning. The plasmid coding for Dronpa, (pCDNA3 Dronpa) and Dronpa-Vpr was generated by standard PCR cloning. ptetherin-Flag was constructed by inserting the Flag tag after the predicted coiled-coil domain (residue 154) by PCR cloning as previously described [3]. pmEosFP-tetherin contains mEosFP separated by a spacer peptide (*ggglyksglrsra*) from tetherin N-terminus. Plasmid coding for HA-tetherin was previously described in [19]. Details for primers and cloning can be provided upon request. Plasmids coding for tetherin-HA as well as mutants delTM and delGPI [13] were a kind gift from P. Bieniasz (Aaron Diamond AIDS Research Center, NY).

### Transfections, virus production and infectivity assay

293T cells were transfected using a standard calcium-phosphate-based technique. HeLa cells were transfected using Lipofectamine 2000 (Invitrogen) or Fugene HD (Roche), according to manufacturer instructions. Infectious HIV-1 particles were produced from 293T cells, filtered through a 0.45 µm filter and concentrated through 20% sucrose by centrifugation [6]. Viral titer was determined by applying limiting dilutions of filtered supernatants from producer cells on HeLa TZMbl indicator cells for 48h. Cells were fixed in 1% formaldehyde and stained for β-lactamase expression with 5-bromo-4-chloro-3-indolyl-b-D-galactoside (X-gal).

### Protein and immunofluorescence analysis

Enzymatic removal of surface virions was performed by incubating transfected 293T cells 36 h post transfection with 1 U/ML PI-PLC (Invitrogen) in DMEM or 0.2–50 µg/ML subtilisin A (Sigma-Aldrich) in stripping buffer [13] for 1 h at 37°C followed by filtration and concentration through 20% sucrose. Viral pellets and cellular lysates were lysed in RIPA with protease inhibitor (Sigma) and subjected to standard SDS Page/Western blot analysis. Proteins were detected by mouse monoclonal antibodies against Dronpa (Amalgaam), HIV-1 p24CA (183-H12-5C), mature HIV-1 p17MA (4C9, kind gift from M. Marsh), HA tag (Roche), GFP (Miltenyi biotec), PCNA (Oncogene Research Products) or rabbit polyclonal antibodies against tetherin and Vpu made by K. Strebel (obtained through NIH AIDS Research and Reference Reagent Program).

Recombinant mEosFP was purified from *E. coli* BL21 DE3 transformed with pQE32 mEosFP after 5 h culture at 37°C in presence of 100 µM IPTG as described in [40]. Purified protein was desalted using PD10 columns (GE Healthcare) and protein content was determined using BCA (Thermo Scientific) against known concentrations of bovine serum albumin. For single-molecule characterization 1 nM mEosFP was prepared in 1% PVA and spin-coated on clean coverslips for 2 min at 3000 rpm.

For immunofluorescence analysis, HeLa cells or viral particles on coverslips were fixed with 3% paraformaldehyde and incubated under standard conditions with mouse monoclonal antibodies against HA-tag (Covance), Flag-tag (Sigma), HIV-1 p24CA, mature HIV-1 p17MA, mouse polyclonal antibody against tetherin (kindly provided by Chugai Pharmaceutical Co., Ltd, Kanagawa, Japan) or human monoclonal antibody against HIV-1 gp120 (2G12, obtained through NIH AIDS Research and Reference Reagent Program). Alexa Fluor 647-conjugated secondary antibodies and Alexa Fluor 647-conjugated Cholera toxin subunit B were purchased from Invitrogen. Super-resolution microscopy was performed on cells and viruses in chambered coverglass (LabTek) with freshly prepared switching buffer [24].

### Super-resolution microscopy

Super-resolution microscopy was performed on an inverted microscope (Olympus IX-71) equipped with an TIRF oil objective (60x, NA 1.6, Olympus) and two simultaneously acquiring cooled Electron Multiplying-CCD (ImagEM, Hamamatsu). Dronpa was excited with 1–5 kW/cm^2^ of 491 nm laser (Cobolt), mEosFP with 0.4 kW/cm^2^ of 561 nm (Cobolt) and Alexa Fluor 647 with 0.3 kW/cm^2^ of 644 nm (Spectra Physics) diode pumped solid state lasers. Photoactivation of photoactivatable proteins was performed with a 405 nm laser (Cube, coherent) using stroboscopic illumination [31] or continuous illumination with 0.2–1.8 W/cm^2^ obtained with an optical acoustic modulator (AA Opto-Electronics). Alexa Fluor 647 was activated by 491 nm (Cobolt) or 532 nm (JP Uniphase) laser diode pumped solid state laser.

For the simultaneous measurement of Dronpa and Alexa Fluor 647, the laser lines (405, 491 and 64 nm) were combined using a 405 (zt405rdc, Chroma) and a 505 dichroic (505DCLP, Chroma) and further guided onto the sample through the same dichroic mirror (z488/633rdc, Chroma). When measuring mEosFP and Alexa Fluor 647 simultaneously, the laser lines (405, 532, 561 and 644 nm) were combined using a 405 (zt405rdc, Chroma), a 532 (z532rdc, Chroma) and a 561 dichroic (z561rdc, Chroma). The four laser lines were then guided onto the sample through the same dichroic mirror (z561/644rdc, Chroma).

Emission from TIRF illuminated sample was collected by the same objective and split by 650 long pass dichroic mirror (z650rdc, Chroma). Additional filters were used to remove excitation light and signal from other channels, namely 527/30 band pass for Dronpa (HQ527/30m, Chroma), 570 long pass and 607/67 band pass for mEosFP (HQ570LP and HQ607/67, Chroma), 665 long pass and 700/71 band pass for Alexa Fluor 647 (HQ665LP and HQ700/71x, Chroma). The images were acquired with a final maximum field of view of ca. 41×41 µm^2^ (80×80 nm^2^ per pixel) with a frame rate of 10–20 Hz. In order to reduce the background and the crosstalk between the two channels, the excitation of the two fluorophores and data acquisition were synchronized using 2 mechanical shutters (NewPort). No additional stabilization of the system was required. Analysis of image sequences was performed with a homemade MATLAB routine for single molecule localization [31].

Registration mapping for two-color imaging was performed by a homemade Matlab routine. Briefly, fields of 100–400 fluorescent beads (100 nm, Tetraspek, Invitrogen) were recorded as fiducial markers in both channels. Individual bead positions were determined by Gaussian fitting for both channels, assigned automatically and used to calculate a local weighted mean mapping as described in [32] and detailed in Figure S3.

### Cluster and cocluster analysis

For the determination of the cluster sizes, individual clusters were identified as discrete accumulations of 100 or more single molecule localizations within a fixed radius of 100–200 nm. The sizes were determined as 4 sigma of Gaussian function fitted to the distribution of localizations. Ripley's L function and bivariant Ripley's L function were determined from 4×4 µm^2^ field of view as described [66].

### Counting of mEosFP molecules

For counting the number of mEosFP molecules per cluster images were acquired using continuous illumination with 0.4 kW/cm^2^ of 561 nm to excite the red form of mEosFP and 0.5–1.8 W/cm^2^ 405 nm laser light for photoactivation. Centers of individual clusters of mEosFP-tetherin within 100 nm from ENV staining were manually selected in two-color super-resolution images. The intensity of 9 pixels around the cluster position was measured and plotted against time for 15′000 frames. Single molecules were identified in intensity traces when intensity signals increased 10 standard deviations above background [38] using a homemade Matlab routine. Intensity increases within a dark time of 5 s were not considered to exclude blinking and recovery from long-lived dark states of activated mEosFP molecules.

Of note, the number of mEosFP-tetherin molecules represents a lower estimate since photoactivation prior to visualization, incomplete photoactivation during the experiment and missed detection of activated molecules may not be completely excluded.

### Accession numbers

The human tetherin clones used in this study correspond to Swiss-Prot entries Q10589.

## Supporting Information

Figure S1 **Screening photoactivatable fluorescent proteins (PAFP) for HIV-1 Gag labeling and PALM.** (A) Constructs used for PALM: HIV-1 Gag consisting of matrix (MA), capsid (CA), nucleocapsid (NC) and p6 was fused to different photoactivatable proteins, namely Dronpa, PS-CFP2, Dendra2, mKikGR, mEosFP and PAmCherry. Color code refers to standard emission color and gray indicates initial or photoinduced dark state. Numbers indicate wavelengths in nm used for photoactivation or excitation. (B) Western blot analysis (anti-p24CA) of cellular lysates and purified virions from 293T cells transfected with expression plasmids for HIV-1 together with indicated HIV-1 Gag-PAFP. (C) Infectivity of released virus as determined by single cycle replication assay in TZMBL cells and X-gal staining, “no PAFP” represents 100%. Error bars represent standard deviations (n≥4), (D) Labeled virions analyzed by total internal reflection fluorescence microscopy (TIRF, top row) and PALM (bottom row) for comparison purposes, scale bar 200 nm.
(TIF)

Figure S2 **Setup and excitation/detection scheme used for super-resolution microscopy.** Setup, excitation and detection scheme used to measure Dronpa and Alexa Fluor 647 (A) or mEosFP and Alexa Fluor 647 fluorescence (B). Specific dicroic mirrors (DM1-4) and filters (BP1-2) are described in Material and Methods. OAM: acoustic optic modulator, MS1: mechanical shutter synchronized with CCD1, MS2: mechanical shutter synchronized with CCD2.
(TIF)

Figure S3 **Colocalization procedure for two-color super-resolution microscopy.** 100 nm fluorescent beads were used as fiducial markers to correct differences in alignment and chromatic aberrations of detection paths (A,B). Fields of 150–500 beads were imaged simultaneously in Dronpa channel (green) and Alexa Fluor 647 channel (red) and representative part of 2×2 µm is shown. Center positions of beads were determined from Gaussian fitting and corresponding pairs of positions assigned. Localization precision for beads was found 7.6 and 6.1 nm for Dronpa (green) and Alexa Fluor 647 (red) channel, respectively (C), Pairs of center positions were used to calculate a local weighted mean transformation needed to correct images (D,E). Colocalization precision of 17±20 nm (mean ± StD, n = 1857) as measured from distances between bead positions after application of transformation (F).
(TIF)

Figure S4 **Incorporation of PAFP into HIV-1 virions and size histograms.** A) Immunofluorescence analysis (anti-CA, red) of HIV-1 virions containing indicated PAFP (green), scale bar 1 µm. B) Size distribution of Dronpa-Vpr, Gag-Dronpa and Gag-mEosFP in HIV-1 virions from super-resolution imaging and cluster analysis. C) Size distribution of integrase (IN), capsid (CA), matrix (MA) and Envelope (ENV) in HIV-1 virions from super-resolution imaging and cluster analysis.
(TIF)

Figure S5 **Calibration of cluster size analysis.** (A) Examples of simulated fields of 25 circular cluster of different sizes containing 100 random localizations, scale bar 1 µm, (B) Ripley's L analysis of simulated clusters: Note decrease of peaks and shift of maxima towards larger r at larger cluster sizes, (C) Sizes of simulated clusters were estimated using Cluster analysis or Ripley's L maxima, error bars represent StD from>6 fields. (D) Sizes of simulated clusters with varied number of localizations per cluster were estimated using cluster analysis. Error bars represent standard deviations from measurements on six fields. Note that there is no effect of number of localizations on mean cluster size
(TIF)

Figure S6 **Characterisation of tetherin mutants.** (A) 293T cells were transfected with HIV-1 Δvpu and either tetherin-HA, tetherin-HA delTM or tetherin-HA delGPI without or with Vpu as indicated and infectious output was determined on HeLa indicator cells. Error bars represent range of duplicate titrations. (B) Western blot analysis of cell lysates from A) was performed for pr55Gag, tetherin, Vpu, GFP as transfection control and PCNA as loading control. Note that data shown in panel A and B were obtained under identical conditions as Figure 2 B,C. (C) 293T cells were transfected with HIV-1 Δvpu and either HA-tetherin or mEosFP-tetherin. Virions that were constitutively released (const.) or released following incubation of cells with subtilisin A (SubtA) were pelleted through sucrose. Virions and corresponding cell lysates were analyzed by quantitative Western blotting with anti-HA and anti-p24CA antibodies. Numbers below each lane indicate integrated densities of p24CA signal in arbitrary units. Sizes of molecular weight markers in B and C are shown in kilodaltons. (D) Histogram of number of mEosFP-tetherin clusters per HIV-1 budding site. Error bars represent standard deviations from quantification in 5 individual super-resolution images with a total of 550 tetherin-positive HIV-1 budding sites analyzed.
(TIF)

Figure S7 **Single molecule photo-physical characterization of mEosFP.** (A) SDS-PAGE and Coomassie staining of 1 or 4 µg of purified 6xHis-tagged mEosFP. Sizes of molecular weight markers are shown in kilodaltons. The predicted size of 6xHis mEosFP is 31 kDa and lower molecular weight bands represent mEosFP cleavage products associated with premature photoactivation. (B) Representative single molecule traces of mEosFP molecules in 1% PVA under 0.4 kW/cm^2^ 561 nm excitation and pulsed (red) or continuous (black) photoactivation (PA) by 405 nm light (0.2–0.5 kW/cm^2^). Traces comprise 15 000 frames acquired with 50 ms/frame. (C) Determination of dark times (td) of the photoactivated red form of mEosFP under continuous photoactivation. Number of reactivation events per single molecule trace of (total of 15000 frames with 50 ms/frame) were determined from 25–50 traces for different t_d_ and 405 nm intensities. (D) Histogram of number of reactivation events per single molecule trace acquired under pulsed photoactivation (PA) or continuous PA using 405 nm light (0.5–1.8 W/cm^2^) and analyzed using t_d_ =  or 5 s. (E) Effect of excitation light on quantification of single mEosFP molecules in 1% PVA. The red form of mEosFP was detected under continuous 561 nm excitation (0.4 kW/cm^2^). Continuous photoactivation by 405 nm light (1.8 W/cm^2^) was switched on after 20–500 s corresponding to usual acquisition times used. Representative regions of super-resolution images are shown. Scale bar 1 µm. (F) Cluster analysis revealed similar number of detected mEosFP molecules following different exposure intervals with 561 nm excitation light as in (E). Scale bar 1 µm
(TIF)
