# Supplementary figures and images for: Quantitative Multicolor Super-Resolution Microscopy Reveals Tetherin HIV-1 Interaction
Source: PLoS Pathog. 2011 Dec 15;7(12):e1002456. doi: 10.1371/journal.ppat.1002456 (PMC3240612; doi:10.1371/journal.ppat.1002456)

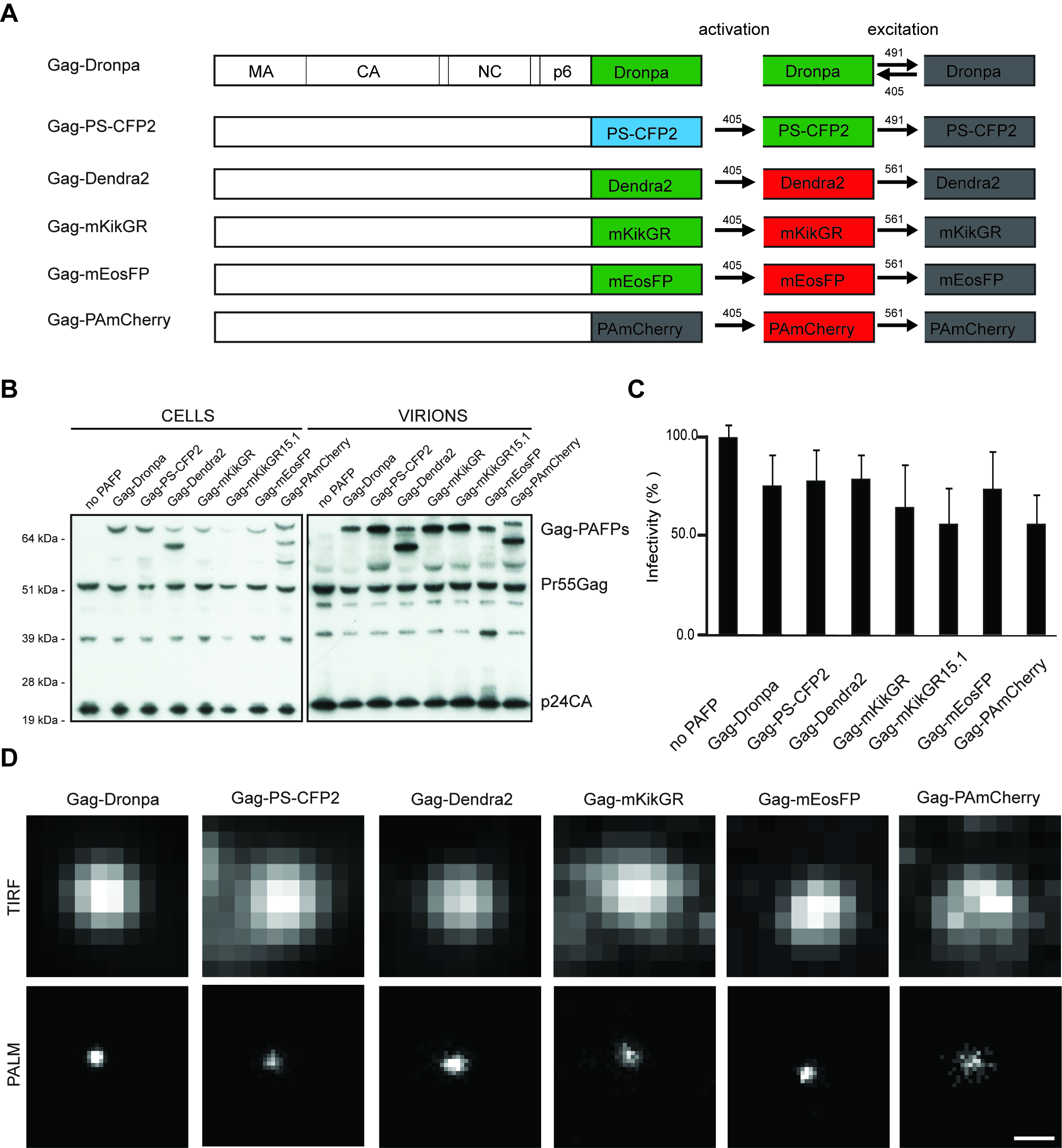

Supplement: Figure S1 — Screening photoactivatable fluorescent proteins (PAFP) for HIV-1 Gag labeling and PALM. (A) Constructs used for PALM: HIV-1 Gag consisting of matrix (MA), capsid (CA), nucleocapsid (NC) and p6 was fused to different photoactivatable proteins, namely Dronpa, PS-CFP2, Dendra2, mKikGR, mEosFP and PAmCherry. Color code refers to standard emission color and gray indicates initial or photoinduced dark state. Numbers indicate wavelengths in nm used for photoactivation or excitation. (B) Western blot analysis (anti-p24CA) of cellular lysates and purified virions from 293T cells transfected with expression plasmids for HIV-1 together with indicated HIV-1 Gag-PAFP. (C) Infectivity of released virus as determined by single cycle replication assay in TZMBL cells and X-gal staining, “no PAFP” represents 100%. Error bars represent standard deviations (n≥4), (D) Labeled virions analyzed by total internal reflection fluorescence microscopy (TIRF, top row) and PALM (bottom row) for comparison purposes, scale bar 200 nm. (TIF) [file ppat.1002456.s001.tif]

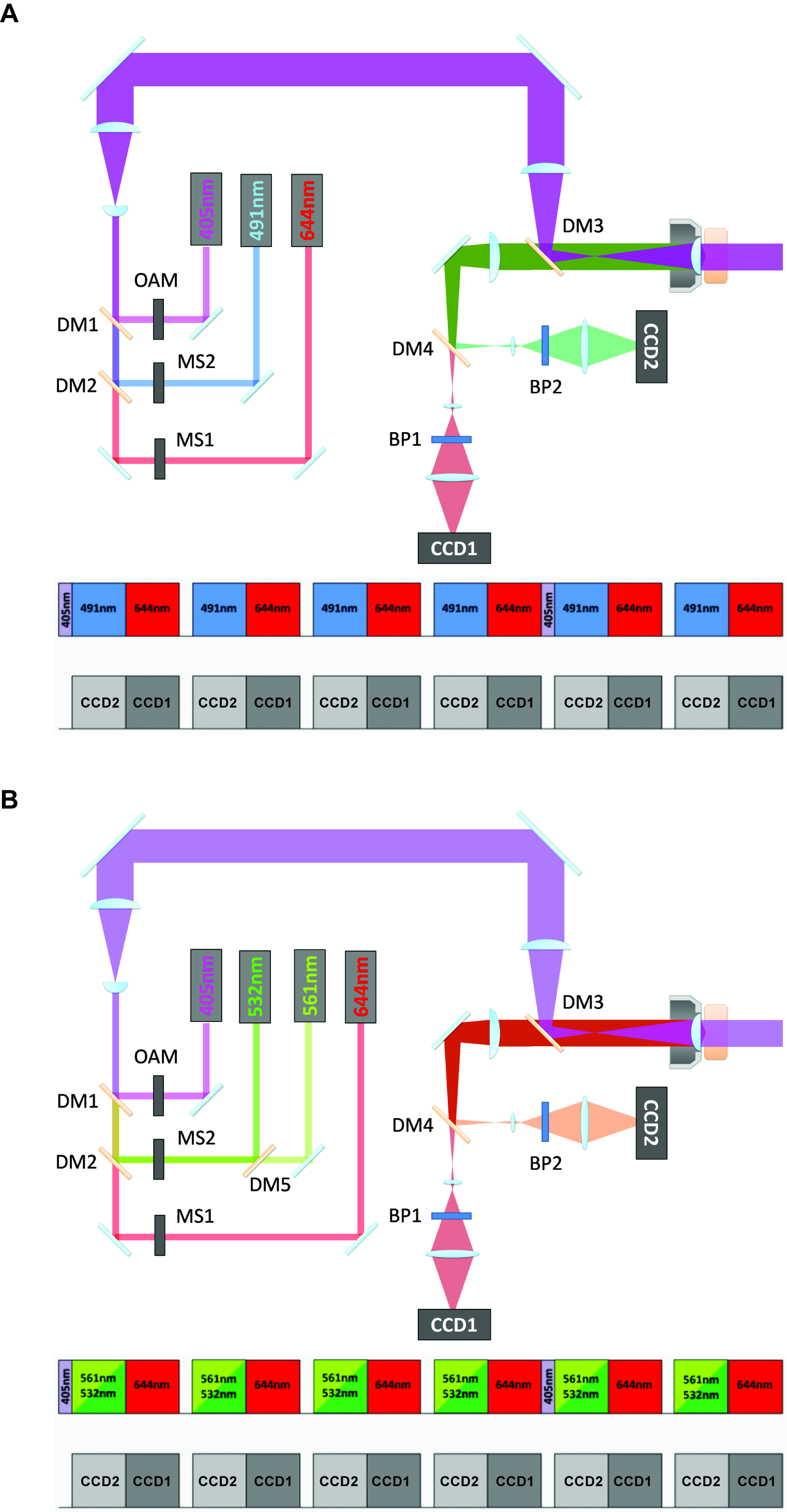

Supplement: Figure S2 — Setup and excitation/detection scheme used for super-resolution microscopy. Setup, excitation and detection scheme used to measure Dronpa and Alexa Fluor 647 (A) or mEosFP and Alexa Fluor 647 fluorescence (B). Specific dicroic mirrors (DM1-4) and filters (BP1-2) are described in Material and Methods. OAM: acoustic optic modulator, MS1: mechanical shutter synchronized with CCD1, MS2: mechanical shutter synchronized with CCD2. (TIF) [file ppat.1002456.s002.tif]

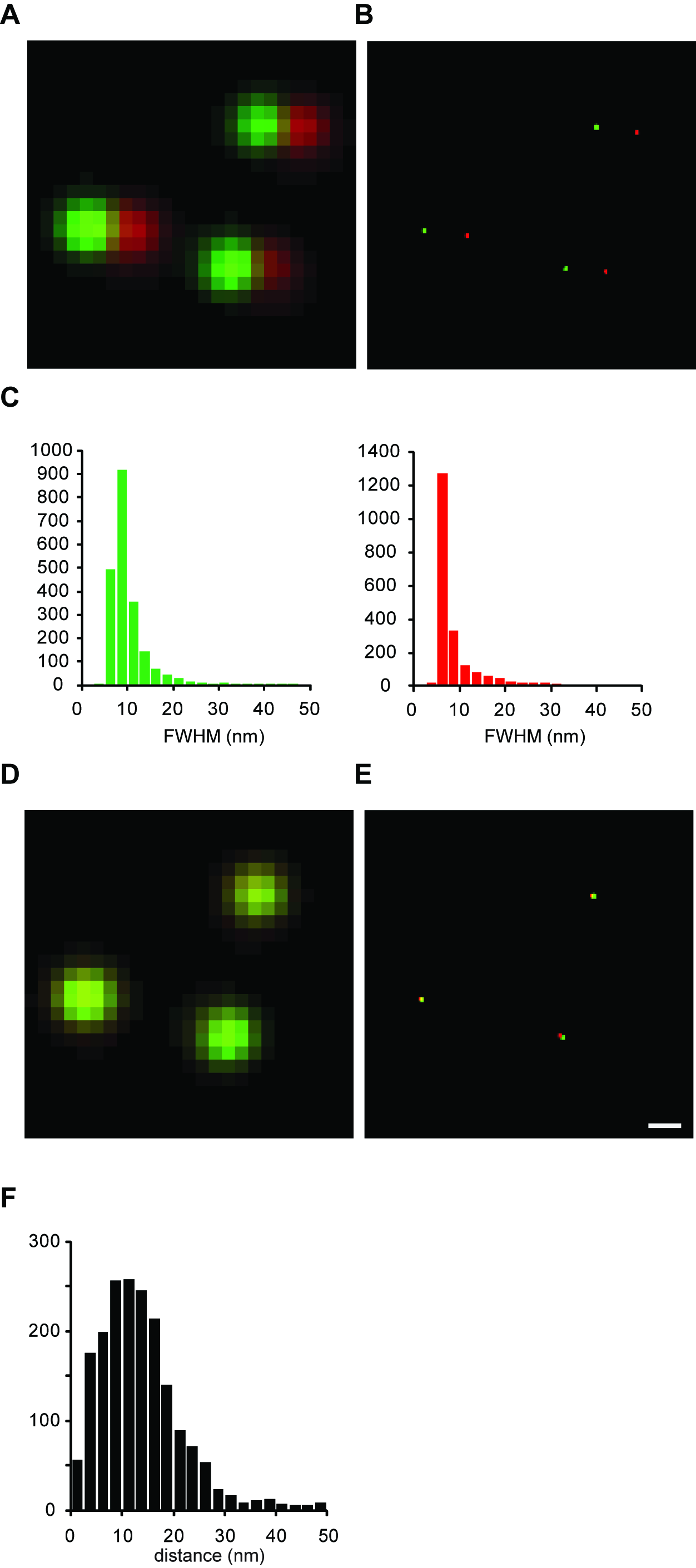

Supplement: Figure S3 — Colocalization procedure for two-color super-resolution microscopy. 100 nm fluorescent beads were used as fiducial markers to correct differences in alignment and chromatic aberrations of detection paths (A,B). Fields of 150–500 beads were imaged simultaneously in Dronpa channel (green) and Alexa Fluor 647 channel (red) and representative part of 2×2 µm is shown. Center positions of beads were determined from Gaussian fitting and corresponding pairs of positions assigned. Localization precision for beads was found 7.6 and 6.1 nm for Dronpa (green) and Alexa Fluor 647 (red) channel, respectively (C), Pairs of center positions were used to calculate a local weighted mean transformation needed to correct images (D,E). Colocalization precision of 17±20 nm (mean ± StD, n = 1857) as measured from distances between bead positions after application of transformation (F). (TIF) [file ppat.1002456.s003.tif]

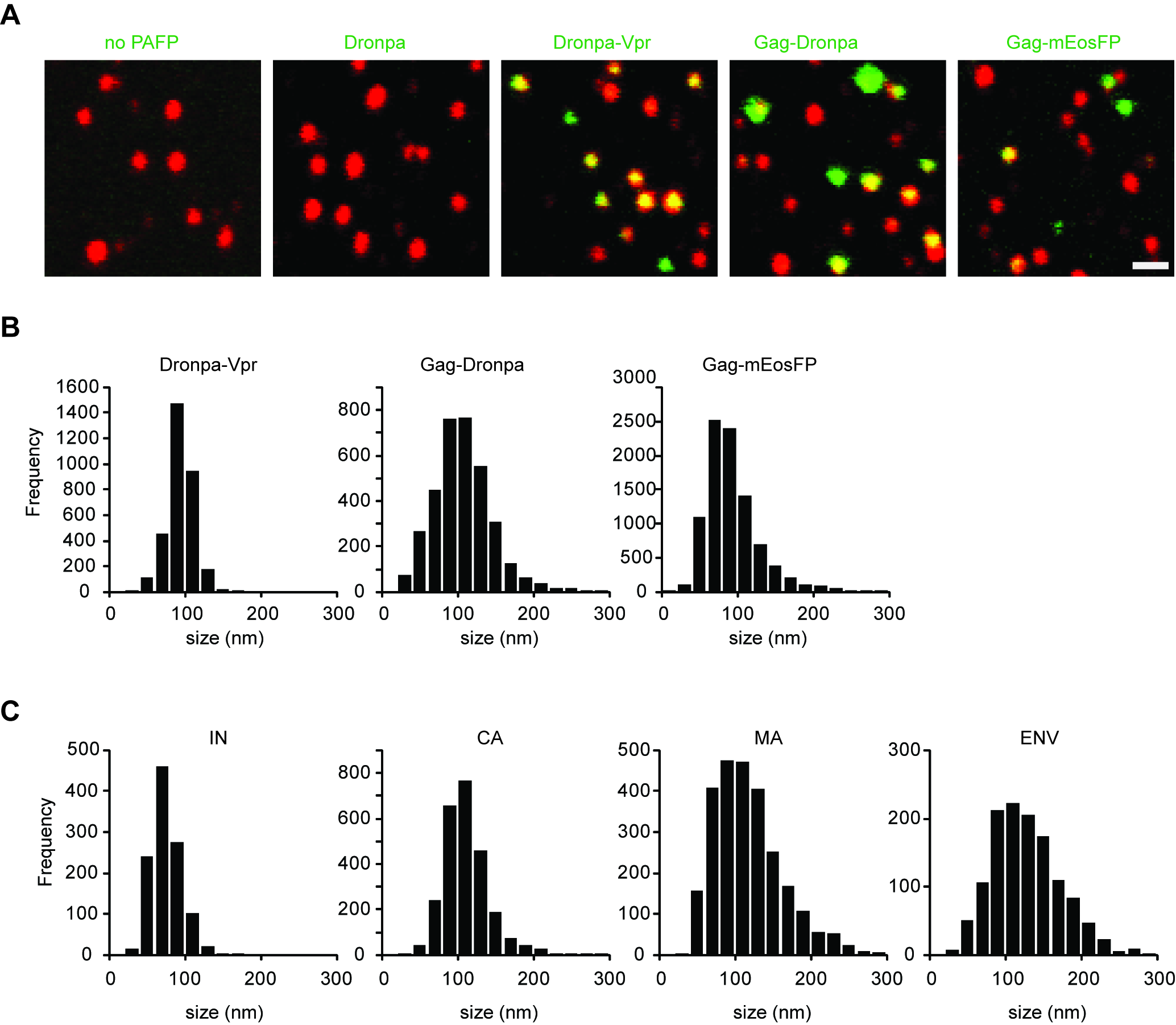

Supplement: Figure S4 — Incorporation of PAFP into HIV-1 virions and size histograms. A) Immunofluorescence analysis (anti-CA, red) of HIV-1 virions containing indicated PAFP (green), scale bar 1 µm. B) Size distribution of Dronpa-Vpr, Gag-Dronpa and Gag-mEosFP in HIV-1 virions from super-resolution imaging and cluster analysis. C) Size distribution of integrase (IN), capsid (CA), matrix (MA) and Envelope (ENV) in HIV-1 virions from super-resolution imaging and cluster analysis. (TIF) [file ppat.1002456.s004.tif]

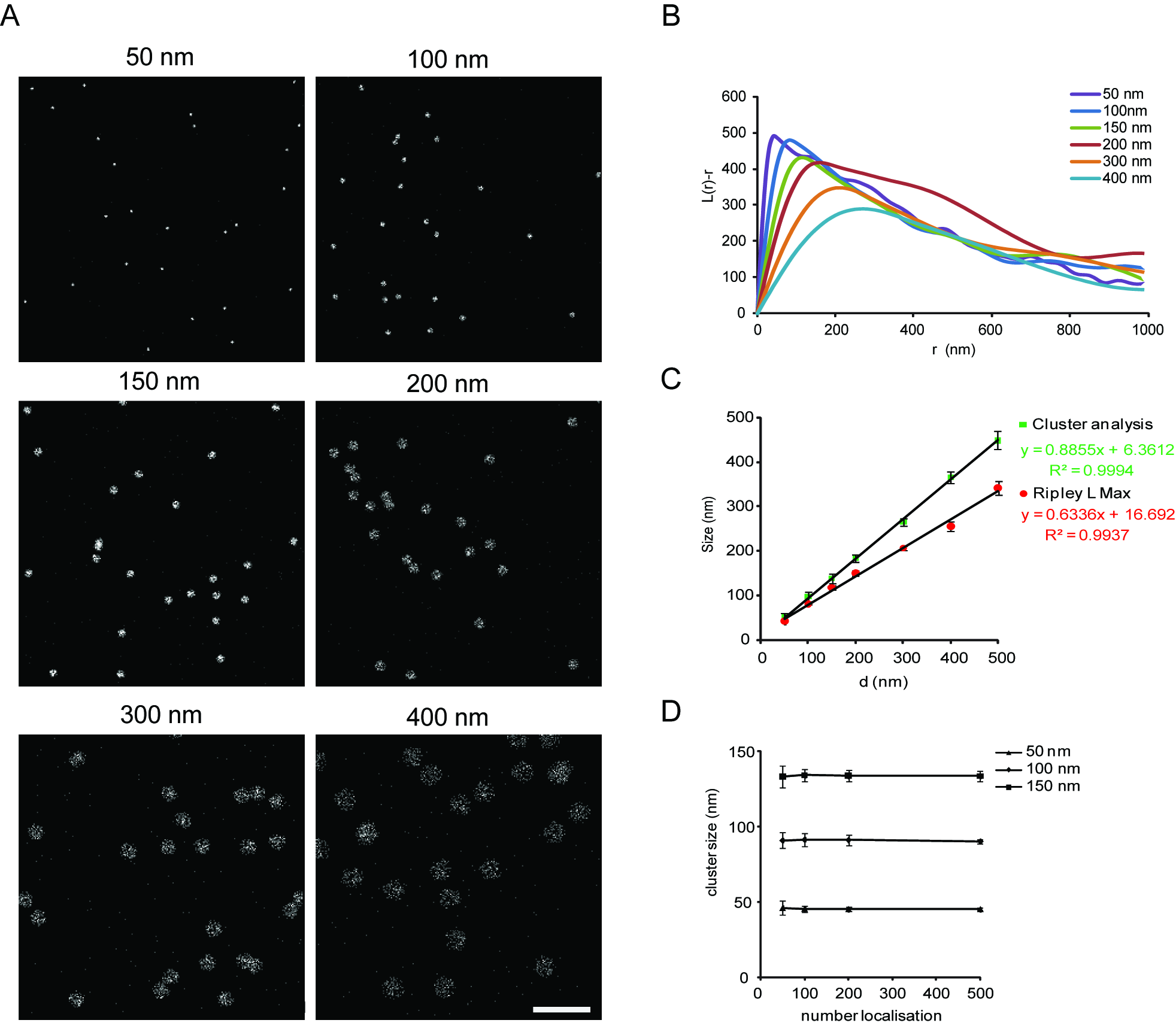

Supplement: Figure S5 — Calibration of cluster size analysis. (A) Examples of simulated fields of 25 circular cluster of different sizes containing 100 random localizations, scale bar 1 µm, (B) Ripley's L analysis of simulated clusters: Note decrease of peaks and shift of maxima towards larger r at larger cluster sizes, (C) Sizes of simulated clusters were estimated using Cluster analysis or Ripley's L maxima, error bars represent StD from>6 fields. (D) Sizes of simulated clusters with varied number of localizations per cluster were estimated using cluster analysis. Error bars represent standard deviations from measurements on six fields. Note that there is no effect of number of localizations on mean cluster size (TIF) [file ppat.1002456.s005.tif]

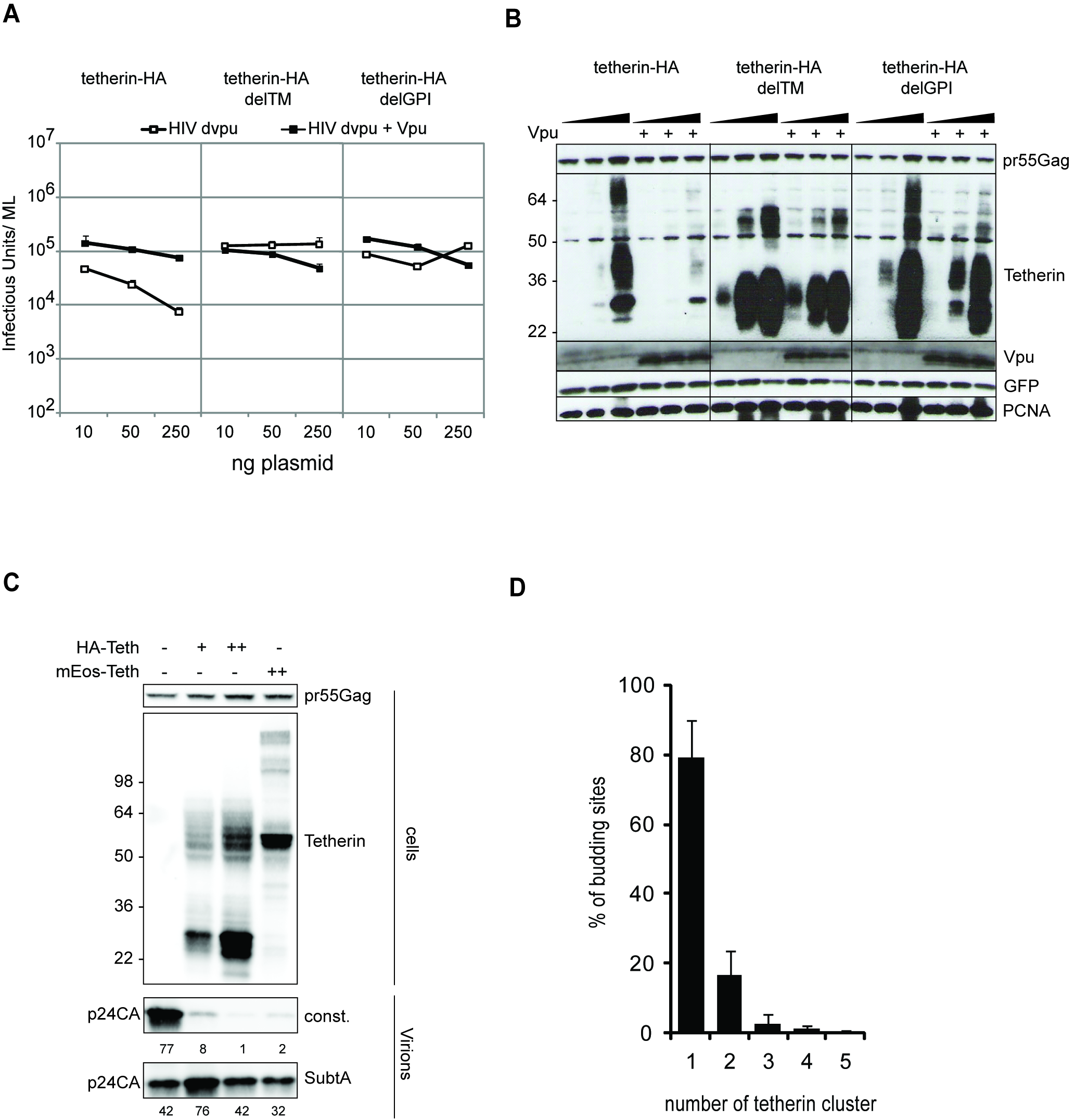

Supplement: Figure S6 — Characterisation of tetherin mutants. (A) 293T cells were transfected with HIV-1 Δvpu and either tetherin-HA, tetherin-HA delTM or tetherin-HA delGPI without or with Vpu as indicated and infectious output was determined on HeLa indicator cells. Error bars represent range of duplicate titrations. (B) Western blot analysis of cell lysates from A) was performed for pr55Gag, tetherin, Vpu, GFP as transfection control and PCNA as loading control. Note that data shown in panel A and B were obtained under identical conditions as Figure 2 B,C. (C) 293T cells were transfected with HIV-1 Δvpu and either HA-tetherin or mEosFP-tetherin. Virions that were constitutively released (const.) or released following incubation of cells with subtilisin A (SubtA) were pelleted through sucrose. Virions and corresponding cell lysates were analyzed by quantitative Western blotting with anti-HA and anti-p24CA antibodies. Numbers below each lane indicate integrated densities of p24CA signal in arbitrary units. Sizes of molecular weight markers in B and C are shown in kilodaltons. (D) Histogram of number of mEosFP-tetherin clusters per HIV-1 budding site. Error bars represent standard deviations from quantification in 5 individual super-resolution images with a total of 550 tetherin-positive HIV-1 budding sites analyzed. (TIF) [file ppat.1002456.s006.tif]

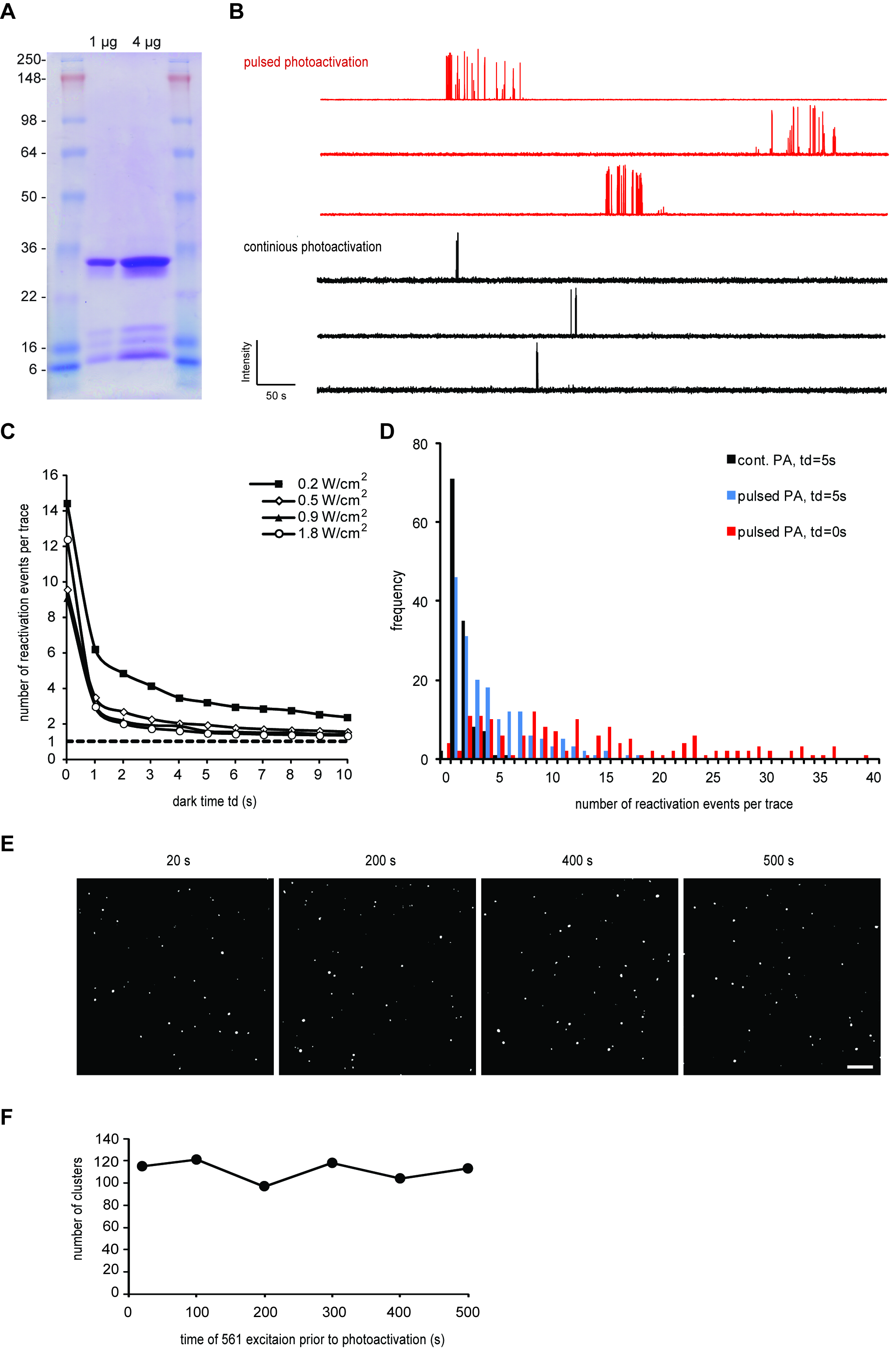

Supplement: Figure S7 — Single molecule photo-physical characterization of mEosFP. (A) SDS-PAGE and Coomassie staining of 1 or 4 µg of purified 6xHis-tagged mEosFP. Sizes of molecular weight markers are shown in kilodaltons. The predicted size of 6xHis mEosFP is 31 kDa and lower molecular weight bands represent mEosFP cleavage products associated with premature photoactivation. (B) Representative single molecule traces of mEosFP molecules in 1% PVA under 0.4 kW/cm2 561 nm excitation and pulsed (red) or continuous (black) photoactivation (PA) by 405 nm light (0.2–0.5 kW/cm2). Traces comprise 15 000 frames acquired with 50 ms/frame. (C) Determination of dark times (td) of the photoactivated red form of mEosFP under continuous photoactivation. Number of reactivation events per single molecule trace of (total of 15000 frames with 50 ms/frame) were determined from 25–50 traces for different td and 405 nm intensities. (D) Histogram of number of reactivation events per single molecule trace acquired under pulsed photoactivation (PA) or continuous PA using 405 nm light (0.5–1.8 W/cm2) and analyzed using td = or 5 s. (E) Effect of excitation light on quantification of single mEosFP molecules in 1% PVA. The red form of mEosFP was detected under continuous 561 nm excitation (0.4 kW/cm2). Continuous photoactivation by 405 nm light (1.8 W/cm2) was switched on after 20–500 s corresponding to usual acquisition times used. Representative regions of super-resolution images are shown. Scale bar 1 µm. (F) Cluster analysis revealed similar number of detected mEosFP molecules following different exposure intervals with 561 nm excitation light as in (E). Scale bar 1 µm (TIF) [file ppat.1002456.s007.tif]
